# Supplementary material for: Ancient DNA study reveals HLA susceptibility locus for leprosy in medieval Europeans
Source: Nat Commun. 2018 May 1;9:1569. doi: 10.1038/s41467-018-03857-x (PMC5931558; doi:10.1038/s41467-018-03857-x)
Supplement: Supplementary file 1 — Supplementary Information [file 41467_2018_3857_MOESM1_ESM.docx]

Supplementary Information

**Ancient DNA study reveals HLA susceptibility locus for leprosy in medieval Europeans**

Krause-Kyora et al.

Supplementary Notes

Note 1. Archaeological sites and specimens analyzed in this study

In the current study, human skeletal specimens were obtained from five medieval cemeteries in Denmark and present-day northern Germany (see cemetery descriptions below). Osteological information (sex, age at death, leprosy status) of the individuals was collected for Odense/St. Jørgen, Revshale and Tirup, whereas data was already available for Ribe and Schleswig/Rathausmarkt from previously published or currently ongoing studies^1,2^.

Leprosy is a slowly evolving chronic disease which leaves distinct lesions on the skeleton that can be reliably diagnosed^3,4^. Extensive osteological analyses have provided considerable evidence for leprosy in skeletal remains from both leprosaria and ordinary parish cemeteries in Denmark. The skeletal sampling strategy used in this study was based upon such work: individuals who suffered from LL and were buried at the leprosarium cemetery of St. Jørgen in Odense are considered cases in our study. Given the high infection prevalence, people interred at ordinary cemeteries might also have been infected. However, if this was the case, they were affected with a milder form of the disease, as evidenced from osteological analysis. These individuals were here regarded as controls. They were selected from the four sites Ribe, Revshale, Tirup and Schleswig/Rathausmarkt that were geographically close and dated earlier or contemporaneous to Odense/St. Jørgen (see below). The remains of the controls showed no osteological evidence of LL and were all *M. leprae* DNA-negative in the PCR-based pathogen screening.

- 1. *Cemetery description*

Odense, St. Jørgen (Denmark)

This cemetery was associated with the leprosarium of the St. Jørgen church in Odense. It was used for the burial of leprosarium patients between AD 1270 and 1550^5^ (Fig. 1). Using the arm positions of the skeletons as an indicator of relative dating, it has been estimated that most burials took place in the 13^th^ and 14^th^ centuries^2^. Radiocarbon dating was performed on samples from 9 skeletons (C14 dates in 2 sigma range listed in (Supplementary Data 1)). Of the 1127 excavated St. Jørgen skeletons, 85 remains with severe lesions of LL were considered cases in the ancient association study. We processed one tooth per individual and in addition one petrous bone from 17 individuals (Supplementary Data 1).

Ribe (Denmark)

This collection includes skeletons from three different parish cemeteries - Grey Friars^6,7^, Black Friars^8^ and Ribe Cathedral^9,10^ (Fig. 1). The burials were dated to medieval and post-medieval periods. About 1500 skeletons were available, of which 42 (archaeologically dated between AD 1050 and 1600) were examined in this study. One tooth per individual was processed.

Revshale (Denmark)

This rural parish cemetery^11,12^ was used between AD 1100 and 1400 (Fig. 1). Of the 204 excavated remains, 45 specimens were investigated in this study. One tooth per individual was processed.

Tirup (Denmark)

This rural parish cemetery^13^ was in use between c. AD 1150 and 1350 (Fig. 1). About 620 skeletons were available, of which 57 were examined in this study. From this cemetery, one petrous bone per individual was sampled and processed.

Rathausmarkt (Schleswig, Germany)

This parish cemetery in the city of Schleswig^14^ was used from the 11^th^ to 13^th^ centuries (Fig. 1). Of the 259 excavated remains, 79 skeletons were analyzed in this study. One tooth per individual was processed.

*Osteological analyses*

Osteological analyses were performed by Jesper Boldsen, Dorthe Pedersen and Peter Tarp (ABDOU, Odense, Denmark). Sex determination was made on the basis of morphological differences in the skull, the pelvis and in the remaining post cranial skeleton^15^. Age estimation was performed using the ’experience based method’^16^. Leprosy-specific lesions (characteristic patterns of inflammation and deformation) were recorded on the rhinomaxilla (nasal spine, nasal aperture, alveolar process of the maxilla, palate) and the postcranium (fibula, 5^th^ metatarsal). The lesions are described in detail in Boldsen, 2007^17^.

Note 2. PCR-based experiments

Detailed results are available in Supplementary Data 1.

PCR-based pathogen screening

In total, 40 samples from the St. Jørgen cemetery were found positive for *M. leprae* DNA. None of the 223 tested medieval controls showed PCR products for *M. leprae* (Supplementary Data 1).

PCR-based SNP analysis

Sixty-nine of the analysed 85 samples from St. Jørgen and 152 of the 223 controls yielded genotypes. The frequency of the T allele is 28.3% in St Jørgen and 18.4% in the controls (Supplementary Data 1).

PCR-based human mitochondrial DNA analysis

Of the tested 308 skeletons, 297 samples were successfully haplotyped using PCR (success rate = 96.7%) (Supplementary Data 1).

Note 3. Genome-wide analysis of *M. leprae* and metagenome screening

High-throughput sequencing produced between 31 and 1,149 million reads per individual-specific dataset based on UDG-treated libraries (Supplementary Table 2). The quality control criteria were met by 96.4% - 98.9% of the reads during pre-processing. Between 63% and 94.3% of these reads could be merged, indicating presence of small DNA fragments that produced overlapping reads. The 1,048 – 35,561,167 reads per dataset that mapped to the *M. leprae* reference genome TN (0.002% – 9.9% endogenous DNA) had a mean length between 39 and 101 bases. Following the alignment, duplicate reads were identified and removed. We observed a high variability in the proportion of duplicate reads per dataset, ranging from 3.2% to 80.4%, with an average of 23.5%. In ten of our datasets (G34, G154, G404, G427, G507, G533, G722, G749, G1083 and G1149), the TN reference genome was covered to 100% with an average read depth of at least 10-fold, after the removal of duplicates. The coverage in the remaining 60 datasets ranged from 0.2% to 99.7%, with an average read depth of the covered regions ranging from 1.2-fold to 44.6-fold. The damage patterns showed mis-incorporation frequencies of up to 2.6% in the datasets based on UDG-treated libraries (Supplementary Table 5), while the datasets based on non-UDG-treated libraries showed frequencies of up to 12.9% (Supplementary Table 6). The maximal pairwise difference in mis-incorporation frequencies of UDG-treated and non-UDG-treated datasets per individual ranged between 2.88% and 8.78%, indicating that all reads mapping to the TN reference are based on ancient DNA fragments.

Following the duplicate removal, we identified a total of 663 variable positions in all our UDG datasets. The evaluation of the SNP effects showed that no SNP clearly correlated with a possible change in *M. leprae* virulence. Amongst the 663 SNPs identified, 126 were annotated as intergenic, 221 as pseudogenes or unidentified proteins and 155 as located in a stable RNA sequence (Supplementary Data 2). Non-synonymous variants accounted for 51% (339) of all annotated SNPs. Only 20% (67) of the non-synonymous variants fell into the functional categories of information pathways, intermediary metabolism and respiration and cell wall and cell processes.

The *de novo* assembly of mapped reads showed similar results for all datasets with respect to the contig number (113 - 289), the minimal contig size (122b – 212b), the maximal contig size (69,059b – 222,852b), mean contig length (11,209b – 28,379b) and N50 values (17,443b – 47,897b) (Supplementary Table 10). The median number of contigs per dataset showed more variability, ranging from 355b in G507 to 19,598b in G34 (Supplementary Table 10). The length of the generated sequence per dataset was at least 3.19 million bases.

Despite the large differences in quality metrics, the contig reordering relative to the TN reference genome led to a high genome coverage (≥ 97%) for all ten samples (Supplementary Table 11). The number of contigs used in the reordering was very similar for all but one dataset, ranging from 103 – 151, while G1083 was the only outlier with 269 contigs. Nearly all gaps in between the ancient contigs fell in repetitive regions of the modern reference

The descriptive metrics of the *de novo* assembly of shotgun sequence data using megahit showed great variability among all datasets with regard to the number of contigs, contig size and contig length. The number of contigs ranged from 52,084 to 880,484, the maximal contig size from 179,644b to 511,145b, the mean contig length from 537b – 1,164b, the N50 values from 485b to 2424b and the total length of generated sequences from 50.9×106b - 725.7×106b (Supplementary Table 12). Only the minimal contig size of 200b was identical in all ten samples. The described differences in the *de novo* assembly among the datasets did not influence the reordering process. All ten samples covered at least 97% of the reference genome (Supplementary Table 13). The number of used contigs ranged from 120 in G154 to 571 in G507. This difference may be due to sample composition and varying content of *M. leprae* reads.

The metagenome analysis revealed no co-infection in any of the 68 tested tooth samples with available shotgun data. CSV files with taxonomic content were extracted using Megan. The composition of bacterial families was visualized (Supplementary Fig. 2, 3). In all datasets, the relative abundance of endogenous bacteria (e.g. oral bacteria) depended on the number of exogenous bacteria (e.g. soil, plant and water bacteria) in the sample. During sample preparation, the DNA fragments of these contaminants were more likely to be amplified, as their fragments were less degraded than the endogenous aDNA fragments. Thus, exogenous bacteria could be overrepresented, making it difficult to assess the actual composition of the microbiome that was present in the individual while still alive. This observation was expected since teeth were used as starting material and environmental bacteria penetrate them to reach the nutrient source inside (dental pulp and blood vessels). We detected *M. leprae* specific sequences in all 68 tooth samples with the available shotgun data, using both the comparative mapping approach (Malt, Supplementary Table 3) and the direct mapping approach (bowtie2, Supplementary Table 2).

Note 4. Phylogenetic analysis

Phylogenetic analyses were performed using multiple methods to construct trees, revealing a consistent topology for the four major SNP-types (Fig. 4, Supplementary Fig. 4-7) supported by bootstrap values of >90% on all major nodes. Nine of our ten ancient strains grouped together according to their SNP-types (Fig. 4, Supplementary Fig. 4-7). Only G507 clustered with the modern strains S9 and S10 outside the four major SNP-types, forming the recently described branch 0^18^. In the rooted maximum parsimony tree, the strains S9 and S10 fell closest to the out-group *M. avium* 104 and strains of the same SNP type clustered together, analogous to the unrooted trees (Supplementary Fig. 6, Supplementary Data 3).

Note 5. Identification of HLA allele combinations

The 68 aDNA samples used in HTS represented 136 alleles per locus. For the three classical class II HLA *loci*, we were able to call between 46 (33%) and 96 (70%) alleles at the 2-digit level (roughly representing different serotypes) and between 46 (33%) and 79 (57%) alleles at the 4-digit level (representing distinct protein sequences) (Supplementary Table 14, 15)

Note 6. DKMS control samples

DKMS is a non-profit organization recruiting potential volunteer donors for hematopoietic stem cell transplantation. Currently, more than 4.9 million people are registered with DKMS in Germany. Since 2013 donors have been routinely typed at high resolution for HLA-A, -B, -C, -DRB1, -DQB1, and -DPB1 upon registration. DKMS also records self-assessed parentage, categorized by country of origin. The frequency of HLA-DRB1*15:01 was estimated based on donors who identified themselves as ‘German’ and were registered in post code areas starting with 23, 24, or 25. This corresponds roughly to the area of the federal state of Schleswig-Holstein. Frequencies of DRB1~DQB1 haplotypes were obtained from the same sample using Hapl-O-Mat^19^. For the purpose of this analysis, any personal data of respective donors was anonymized.

Note 7. Genome-wide analysis of *H. sapiens* and population genetics

In the alignment, the number of reads per dataset that could be mapped to the human reference genome ranged from 215,766 - 157,386,794, representing an endogenous DNA content of 1.25 - 70.8% (Supplementary Table 7). The mean length of these ranged from 47 - 101 bases. Duplicate reads were identified and removed after the alignment. We observed a high variability in the proportion of duplicate reads per dataset, ranging from 3.0% - 68.2%, with an average of 31.7%. Depending on the size of the dataset and the endogenous DNA content, the coverage of the human reference ranged from 0.4% to 81.1% with an average read depth of up to 5x in the covered regions. The comparison of damage patterns revealed mis-incorporation frequencies of up to 2.6% in UDG- treated and up to 21.9% in non-UDG-treated datasets, indicating that all reads mapping to the human reference originate from ancient DNA fragments (Supplementary Table 8, 9).

The sex of each individual could be estimated based on sequencing data using read densities on the X and Y chromosome. The ratio r=d_Y_ /d_X_ is expected to be close to 0 for females and close to 1 for males. We observed a bimodal distribution of r for the 68 samples from St. Jørgen. Samples with 0.10 ≤ r ≤ 0.24 (median 0.17) were assigned female sex, whereas samples with 0.55 ≤ r ≤ 1.38 (median 0.78) were considered to be males (Supplementary Table 16).

During the estimation of IBD sharing, 2,732,873 non-monomorphic SNPs remained for analysis. Almost all (2,333; 99.4%) of the 2,346 pairs of individuals were un- or very distantly related (π ̂≤0.05), while another three pairs had values of 0.108 - 0.133, which would be consistent with e.g. a first-cousin’s relationship, and another 9 pairs with values between 0.05 and 0.10. A first-degree relationship could thus be excluded with varying degrees of certainty for all (π ̂ median = 0.004, stdev = 0.007) (Supplementary Data 4).

Supplementary Figures


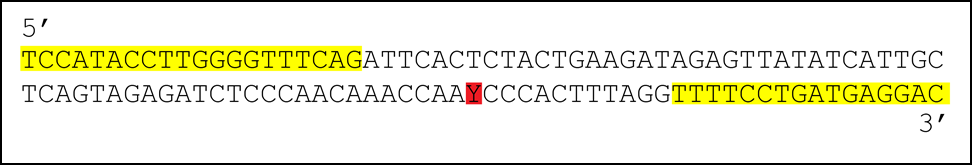


Fig. 1: Sequence surrounding SNP rs3135388

SNP rs3135388 (C🡪T) is marked in red; the primer sequences are highlighted in yellow.


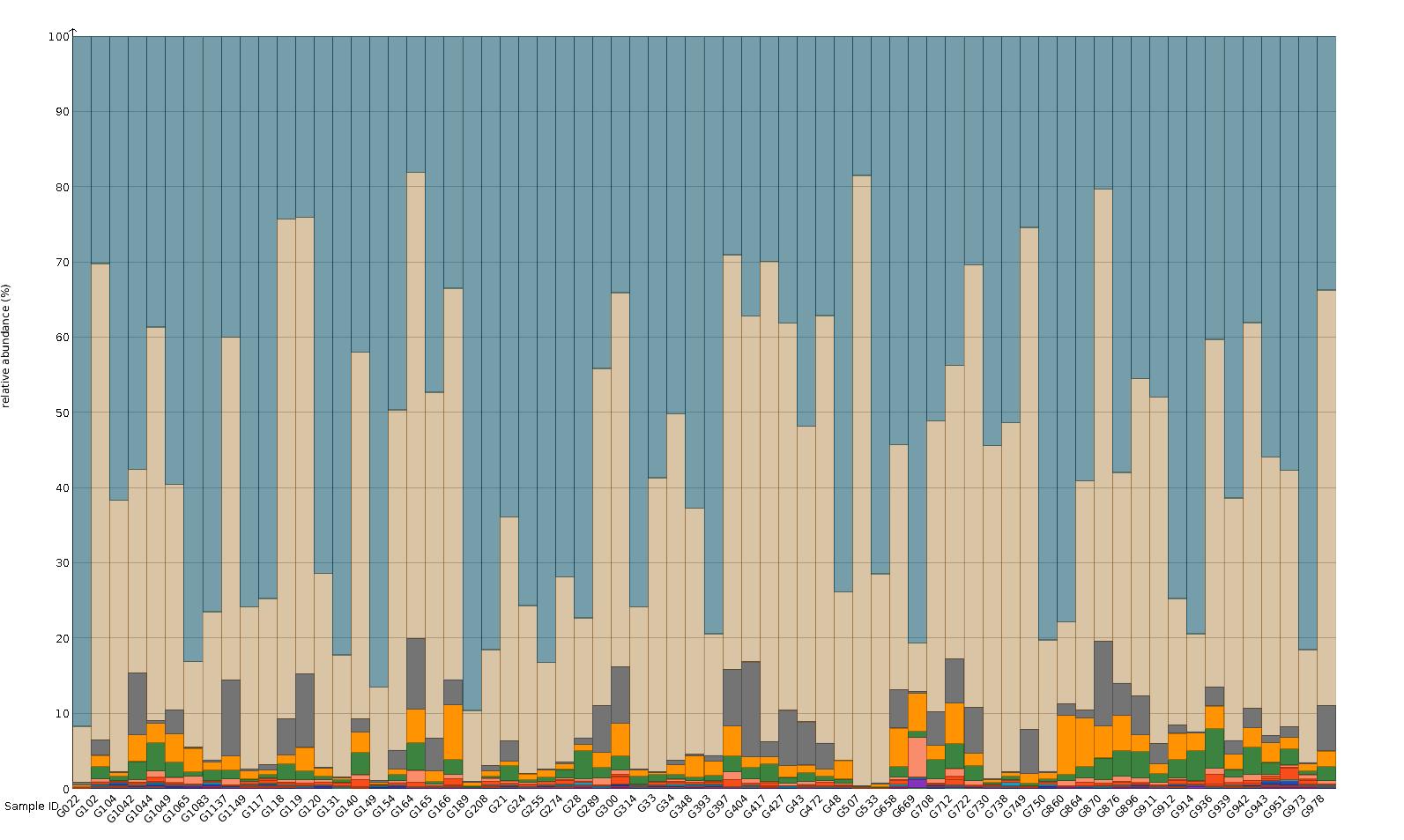


**Fig. 2: Bacteria composition of the 68 tooth samples from St. Jørgen**

Stacked barplot of the bacterial composition. The relative abundance (in %) of bacterial phyla is represented by different colors. Figure was generated with MEGAN^20^.


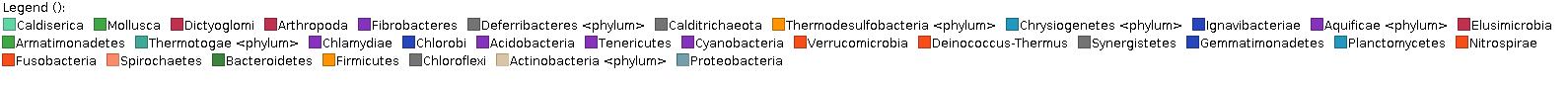


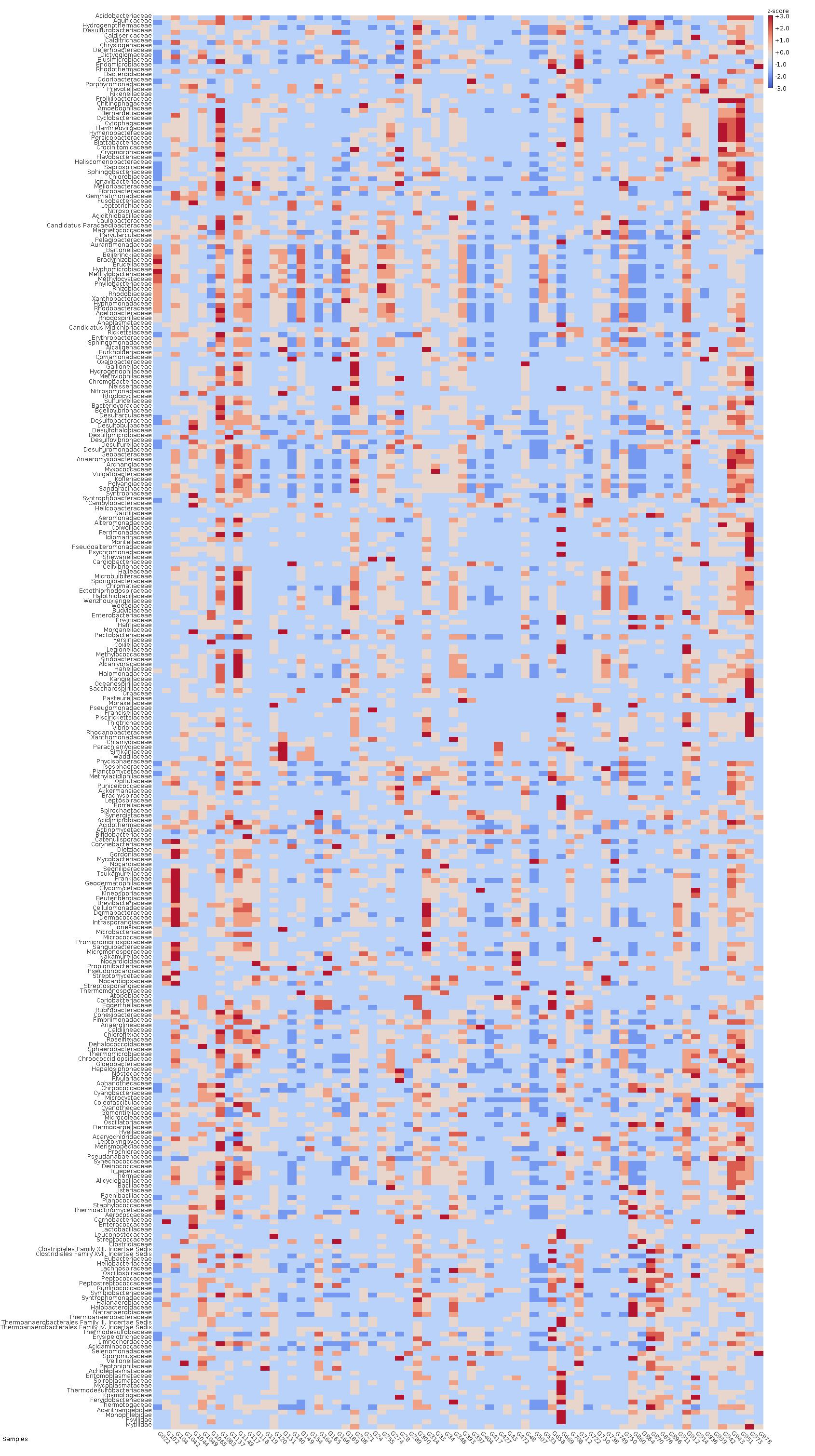


Fig. 3: Bacterial composition of 68 tooth samples from St. Jørgen

Bacterial taxonomic assignments are indicated to the left of the heatmap at the family level. The relative abundance of each bacterial family is color coded as indicated by the key on the right upper corner - Z-scores are centered and normalized, so the color can be interpreted as standard deviations from the mean. Figure was generated with MEGAN^20^.


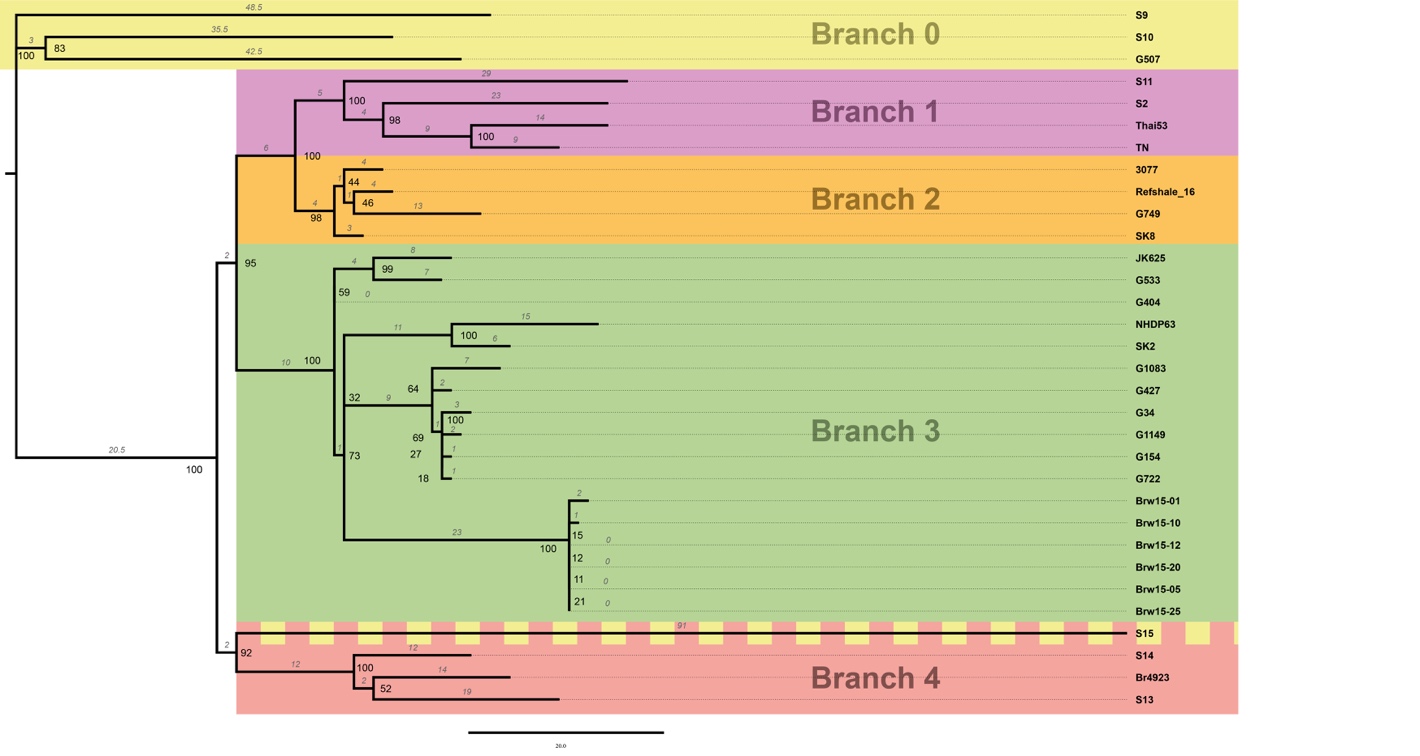


Fig. 4: Maximum parsimony analysis of taxa without outgroup

The evolutionary history was inferred using the maximum parsimony (MP) method. Tree #1 out of 10 most parsimonious trees (length = 547) is shown. The consistency index is (0.912752), the retention index is (0.974806), and the composite index is 0.951639 (0.889756) for all sites and parsimony-informative sites (in parentheses). The percentage of replicate trees in which the associated taxa clustered together in the bootstrap test (500 replicates) is shown next to the branches^21^. The MP tree was obtained using the Subtree-Pruning-Regrafting (SPR) algorithm^22^ with search level 1 in which the initial trees were obtained by the random addition of sequences (10 replicates). The analysis involved 32 nucleotide sequences. All positions containing gaps and missing data were eliminated. There was a total of 534 positions in the final dataset. Evolutionary analyses were conducted in MEGA7^23^.


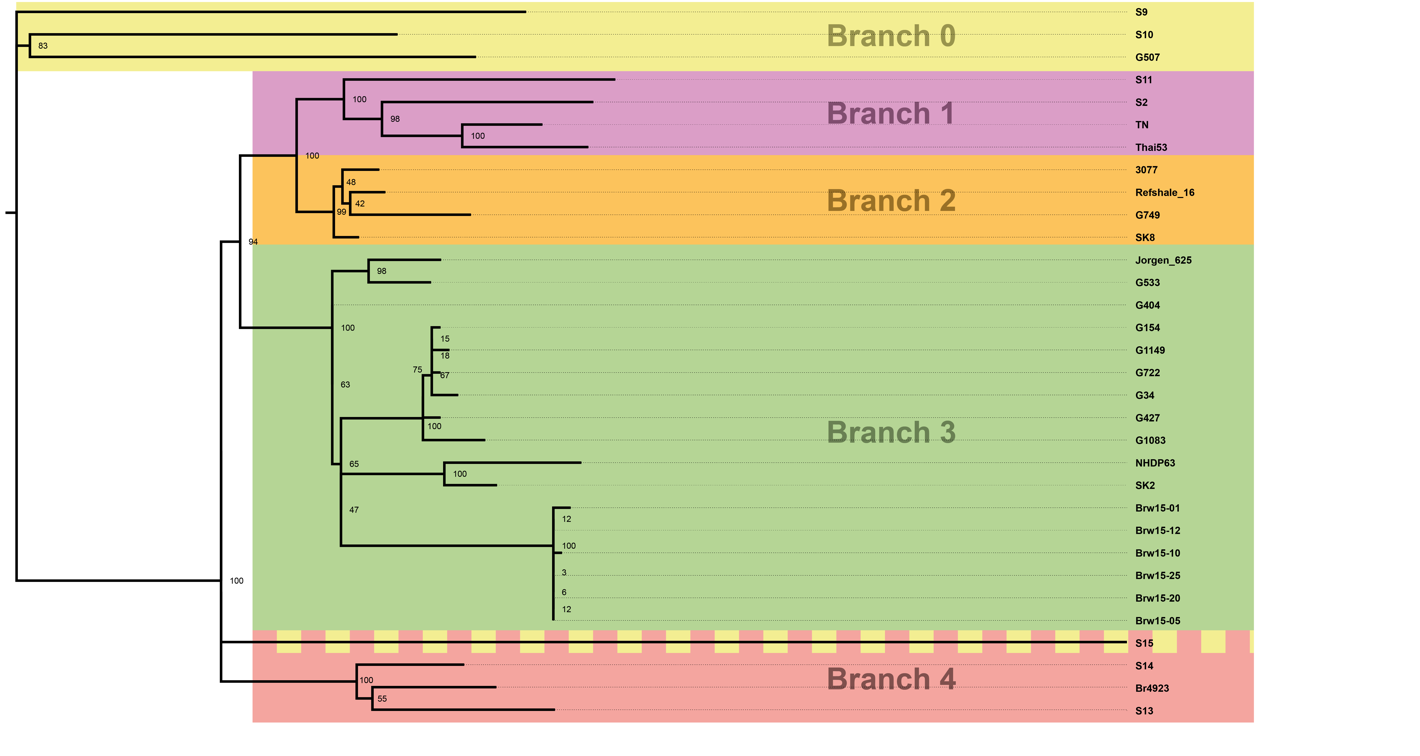


Fig. 5: Molecular phylogenetic analysis by maximum likelihood method

The evolutionary history was inferred by using the maximum likelihood method based on the Tamura 3-parameter model^24^. The tree with the highest log-likelihood (-3394.7419) is shown. The percentage of trees in which the associated taxa clustered together is shown next to the branches. Initial tree(s) for the heuristic search were obtained automatically by applying neighbor-joining and BioNJ algorithms to a matrix of pairwise distances estimated using the maximum composite likelihood (MCL) approach, and then selecting the topology with superior log likelihood value. The tree is drawn to scale, with branch lengths measured in the number of substitutions per site. The analysis involved 32 nucleotide sequences. All positions containing gaps and missing data were eliminated. There was a total of 534 positions in the final dataset. Evolutionary analyses were conducted in MEGA7^23^.


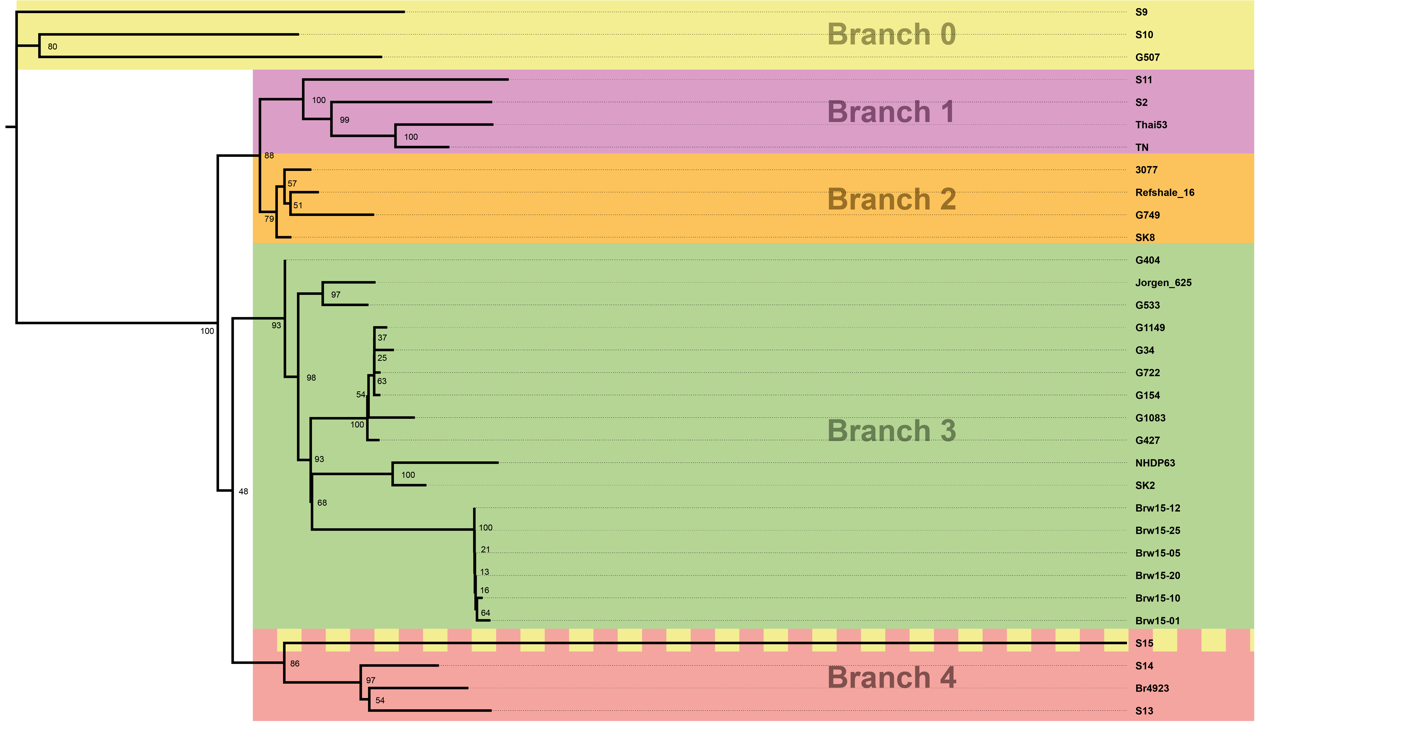


Fig. 6: Evolutionary relationships of taxa

The evolutionary history was inferred using the neighbor-joining method^25^. The optimal tree with the sum of branch length = 1.22434217 is shown. The percentage of replicate trees in which the associated taxa clustered together in the bootstrap test (500 replicates) is shown next to the branches^21^. The tree is drawn to scale, with branch lengths in the same units as those of the evolutionary distances used to infer the phylogenetic tree. The evolutionary distances were computed using the Tamura 3-parameter method^24^ and are in the units of the number of base substitutions per site. The analysis involved 32 nucleotide sequences. All positions containing gaps and missing data were eliminated. There was a total of 534 positions in the final dataset. Evolutionary analyses were conducted in MEGA7^23^.


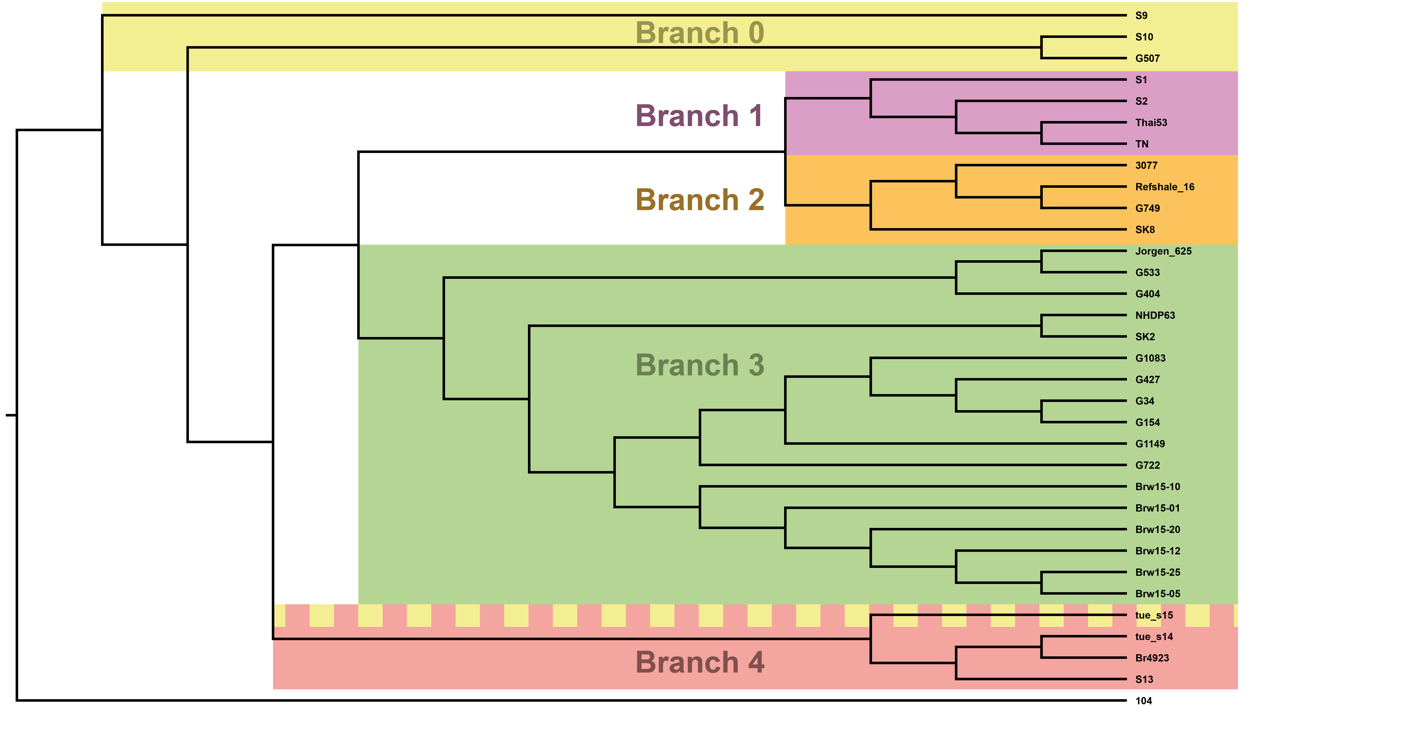


Fig. 7: Maximum parsimony analysis of taxa including outgroup

The evolutionary history was inferred using the maximum parsimony method. Tree #8 out of 9 most parsimonious trees (length = 336616) is shown. The consistency index is (0.771930), the retention index is (0.912752), and the composite index is 0.912646 (0.704580) for all sites and parsimony-informative sites (in parentheses). The percentage of replicate trees in which the associated taxa clustered together in the bootstrap test (500 replicates) is shown next to the branches^21^. The MP tree was obtained using the Subtree-Pruning-Regrafting (SPR) algorithm^22^ with search level 1 in which the initial trees were obtained by the random addition of sequences (10 replicates). The analysis involved 33 nucleotide sequences. All positions containing gaps and missing data were eliminated. There was a total of 336,534 positions in the final dataset. Evolutionary analyses were conducted in MEGA7^23^.


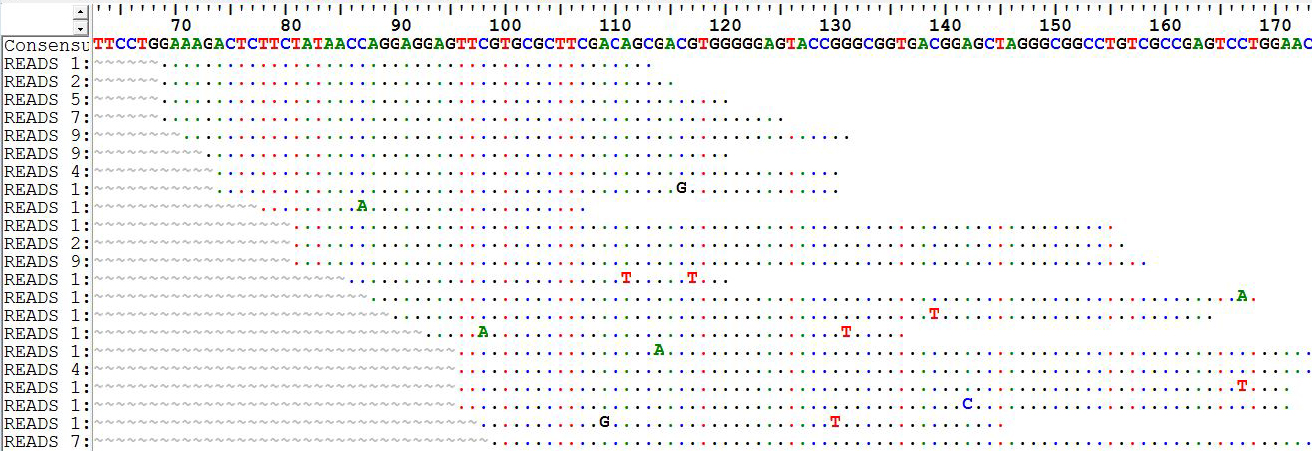


Fig. 8: Manual sorting and filtering of extracted HLA sequences

Visual representation of a sorted group of aligned sequences for a given HLA region in BioEdit^26^, including several reads with PCR/sequencing errors.


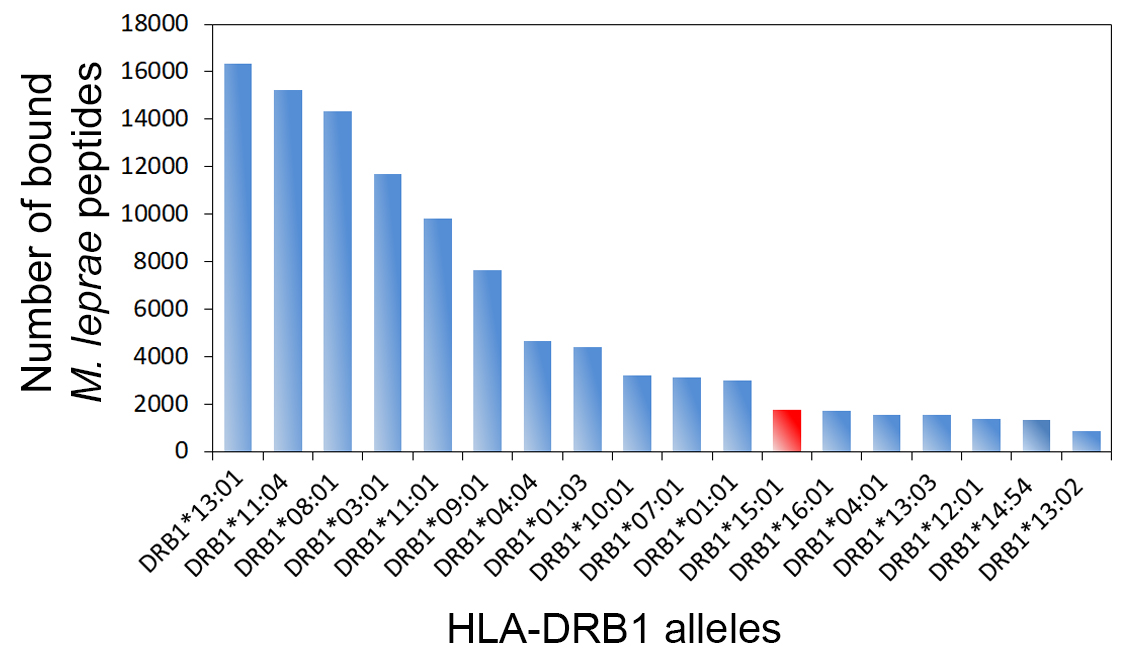


**Fig 9: Prediction of *M. leprae* peptide binding by HLA-DRB1**

Computational prediction of those 15mer peptides of the entire *M. leprae* proteome (N = 516,303 peptides; Ensembl accession: ASM19585v1) that bind to common HLA-DRB1 alleles revealed that DRB1*15:01 (red bar) presents one of the smallest *M. leprae* antigen repertoires. Binding predictions were run for the 18 HLA-DRB1 alleles with an allele frequency of >1% in representative contemporary samples from Schleswig-Holstein/Germany (N = 129,336) and a Danish Minority population from northern Germany (N = 918).

Supplementary Tables

Table 1: PCR products and primer details

| ***Target organism*** | ***Target region*** | ***Amplicon length*** | ***Forward primer***  ***5’ – 3’*** | ***Reverse primer***  ***5’ – 3’*** |
| --- | --- | --- | --- | --- |
| *Human nuclear* | rs3135388 | 108 bp | TCC ATA CCT TGG GGT TTC AG | GTC CTC ATC AGG AAA ACC TAA A |
| *Human mt* | nt 16106-16256 (HV I) | 150 bp | GCC AGC CAC CAT GAA TAT TGT | GCT TTG GAG TTG CAG TTG ATG TGT |
|  | nt 15975-16185 (HV I) | 183 bp | CTC CAC CAT TAG CAC CCA AAG C | TAC TAC AGG TGG TCA AGT AT |
|  | nt 34-287  (HV II) | 253 bp | GGG AGC TCT CCA TGC ATT TGG | TTG TTA TGA TGT CTG TGT GG |
| *M. leprae* | RLEP | 130 bp | TGC ATG TCA TGG CCT TGA GG | CAC CGA TAC CAG CGG CAG AA |
|  | 18kDa | 98 bp | GAG CTG CTC ACC ACA ACA AA | TGT GTC TCC GTT GCA CCT GT |

HV I: hypervariable region I; HV II: hypervariable region II.

Table 2: *M. leprae* alignment of UDG datasets

| ***IID*** | ***# reads aln*** | ***read length aln*** | ***endogenous***  ***DNA [%]*** | | ***duplicate proportion [%]*** | | ***# reads, duplicates***  ***removed*** | ***rmdup cov>=1x [%]*** | ***rmdup cov>=4x [%]*** | ***DP cov*** | ***DP all*** |
| --- | --- | --- | --- | --- | --- | --- | --- | --- | --- | --- | --- |
| *G022* | 15,870 | 91 (63-116) | 0.08 | 10.7 | | 14,171 | | 31.9 | 0.3 | 1.26 | 0.40 |
| *G102* | 5,335 | 45 (35-60) | 0.01 | 14.2 | | 4,575 | | 1.0 | 0.1 | 6.75 | 0.07 |
| *G104* | 3,443 | 48 (38-64) | 0.01 | 15.6 | | 2,907 | | 2.5 | 0.1 | 1.89 | 0.05 |
| *G1042* | 3,119 | 44 (35-61) | 0.01 | 30.8 | | 2,159 | | 0.3 | 0.1 | 10.28 | 0.03 |
| *G1044* | 2,456 | 43 (35-58) | 0.01 | 13.9 | | 2,114 | | 1.1 | 0.1 | 2.91 | 0.03 |
| *G1049* | 2,566 | 43 (35-56) | 0.00 | 26.9 | | 1,876 | | 0.5 | 0.1 | 5.16 | 0.03 |
| *G1065* | 2,097 | 44 (35-56) | 0.00 | 17.3 | | 1,734 | | 0.3 | 0.1 | 8.14 | 0.03 |
| *G1083* | 847,073 | 58 (42-76) | 0.20 | 23.7 | | 646,304 | | 100.0 | 99.4 | 12.19 | 12.19 |
| *G1137* | 125,425 | 77 (53-109) | 0.05 | 29.8 | | 88,075 | | 87.3 | 16.9 | 2.65 | 2.31 |
| *G1149* | 840,703 | 68 (47-76) | 0.17 | 9.8 | | 758,151 | | 100.0 | 99.9 | 15.42 | 15.42 |
| *G117* | 1,048 | 45 (37-59) | 0.00 | 25.1 | | 785 | | 0.3 | 0.1 | 3.87 | 0.01 |
| *G118* | 7,443 | 55 (42-76) | 0.02 | 19.2 | | 6,012 | | 6.8 | 0.1 | 1.65 | 0.11 |
| *G119* | 14,015 | 46 (36-62) | 0.01 | 52.7 | | 6,623 | | 3.1 | 0.1 | 3.33 | 0.10 |
| *G120* | 2,651 | 46 (38-58) | 0.00 | 23.2 | | 2,037 | | 0.3 | 0.1 | 9.11 | 0.03 |
| *G131* | 10,875 | 80 (56-102) | 0.04 | 7.1 | | 10,098 | | 21.0 | 0.1 | 1.23 | 0.26 |
| *G140* | 5,971 | 42 (35-53) | 0.01 | 25.8 | | 4,429 | | 0.4 | 0.1 | 16.14 | 0.06 |
| *G149* | 5,349 | 79 (54-101) | 0.01 | 41.0 | | 3,154 | | 6.2 | 0.1 | 1.29 | 0.08 |
| *G154* | 4,638,029 | 76 (55-76) | 1.53 | 29.3 | | 3,276,845 | | 100.0 | 100.0 | 71.83 | 71.83 |
| *G164* | 20,515 | 52 (40-74) | 0.05 | 29.7 | | 14,416 | | 16.9 | 0.2 | 1.53 | 0.26 |
| *G165* | 12,883 | 56 (42-76) | 0.02 | 22.8 | | 9,945 | | 13.6 | 0.2 | 1.40 | 0.19 |
| *G166* | 14,830 | 45 (35-62) | 0.03 | 42.9 | | 8,465 | | 2.2 | 0.2 | 5.84 | 0.13 |
| *G189* | 77,462 | 82 (59-111) | 0.08 | 36.2 | | 49,453 | | 69.7 | 6.2 | 1.91 | 1.33 |
| *G208* | 354,675 | 58 (43-76) | 0.10 | 11.4 | | 314,168 | | 99.6 | 82.3 | 5.99 | 5.97 |
| *G21* | 2,627 | 44 (35-58) | 0.00 | 17.8 | | 2,159 | | 1.8 | 0.1 | 1.79 | 0.03 |
| *G24* | 6,707 | 55 (43-76) | 0.01 | 15.1 | | 5,693 | | 5.3 | 0.1 | 2.08 | 0.11 |
| *G255* | 1,819 | 47 (40-61) | 0.00 | 25.9 | | 1,348 | | 0.9 | 0.1 | 2.26 | 0.02 |
| *G274* | 1,810 | 47 (38-64) | 0.01 | 17.6 | | 1,492 | | 1.1 | 0.1 | 2.12 | 0.02 |
| *G28* | 1,933 | 46 (38-61) | 0.00 | 25.9 | | 1,432 | | 0.2 | 0.1 | 9.05 | 0.02 |
| *G289* | 5,233 | 65 (47-96) | 0.02 | 5.8 | | 4,928 | | 7.7 | 0.1 | 1.44 | 0.11 |
| *G300* | 3,134 | 42 (34-53) | 0.00 | 10.5 | | 2,804 | | 0.6 | 0.1 | 6.76 | 0.04 |
| *G314* | 1,719 | 43 (35-56) | 0.00 | 9.0 | | 1,565 | | 0.4 | 0.1 | 5.35 | 0.02 |
| *G33* | 173,963 | 71 (47-76) | 0.06 | 9.0 | | 158,242 | | 94.7 | 36.0 | 3.44 | 3.26 |
| *G34* | 2,043,996 | 73 (48-76) | 0.47 | 11.5 | | 1,808,104 | | 100.0 | 100.0 | 37.73 | 37.73 |
| *G348* | 6,583 | 54 (41-75) | 0.02 | 14.0 | | 5,659 | | 7.2 | 0.1 | 1.49 | 0.11 |
| *G393* | 3,078 | 42 (33-53) | 0.00 | 23.0 | | 2,370 | | 0.6 | 0.1 | 4.98 | 0.03 |
| *G397* | 5,219 | 45 (37-61) | 0.01 | 20.5 | | 4,147 | | 2.0 | 0.1 | 3.27 | 0.06 |
| *G404* | 566,204 | 88 (61-102) | 0.20 | 12.6 | | 494,583 | | 100.0 | 99.7 | 13.33 | 13.33 |
| *G417* | 13,527 | 41 (34-52) | 0.02 | 40.1 | | 8,101 | | 0.4 | 0.1 | 26.27 | 0.11 |
| *G427* | 11,623,054 | 101 (80-115) | 2.46 | 77.8 | | 2,578,406 | | 100.0 | 100.0 | 74.02 | 74.02 |
| *G43* | 3,302 | 44 (35-60) | 0.00 | 14.5 | | 2,822 | | 1.9 | 0.1 | 2.29 | 0.04 |
| *G472* | 362,963 | 75 (55-101) | 0.11 | 29.6 | | 255,451 | | 99.7 | 84.0 | 6.27 | 6.25 |
| *G48* | 2,583 | 43 (37-52) | 0.01 | 18.4 | | 2,107 | | 0.3 | 0.1 | 10.31 | 0.03 |
| *G507* | 35,561,167 | 95 (66-106) | 9.88 | 37.3 | | 22,283,858 | | 100.0 | 100.0 | 632.30 | 632.30 |
| *G533* | 933,382 | 98 (68-125) | 1.21 | 25.9 | | 691,444 | | 100.0 | 100.0 | 20.59 | 20.59 |
| *G658* | 118,991 | 67 (47-94) | 0.02 | 24.0 | | 90,468 | | 82.9 | 11.6 | 2.54 | 2.10 |
| *G669* | 23,946 | 52 (40-72) | 0.02 | 25.3 | | 17,887 | | 23.1 | 0.2 | 1.37 | 0.32 |
| *G708* | 3,543 | 44 (35-58) | 0.00 | 17.3 | | 2,930 | | 0.4 | 0.1 | 11.92 | 0.04 |
| *G712* | 7,297 | 39 (31-50) | 0.01 | 14.7 | | 6,227 | | 0.4 | 0.1 | 21.84 | 0.08 |
| *G722* | 1,288,683 | 80 (57-116) | 0.28 | 28.7 | | 918,421 | | 100.0 | 100.0 | 25.07 | 25.07 |
| *G730* | 8,003 | 41 (33-54) | 0.01 | 14.2 | | 6,866 | | 4.2 | 0.1 | 2.30 | 0.10 |
| *G738* | 6,976 | 49 (40-66) | 0.01 | 17.0 | | 5,791 | | 0.9 | 0.1 | 11.01 | 0.10 |
| *G749* | 4,111,522 | 94 (65-119) | 6.00 | 35.9 | | 2,635,897 | | 100.0 | 100.0 | 76.58 | 76.58 |
| *G750* | 1,230 | 41 (32-51) | 0.01 | 6.3 | | 1,152 | | 0.4 | 0.1 | 4.02 | 0.02 |
| *G860* | 2,475 | 49 (40-60) | 0.00 | 20.2 | | 1,974 | | 1.0 | 0.0 | 3.32 | 0.03 |
| *G864* | 1,602 | 40 (31-49) | 0.00 | 10.9 | | 1,428 | | 0.3 | 0.1 | 7.06 | 0.02 |
| *G870* | 4,126 | 39 (31-49) | 0.01 | 23.1 | | 3,172 | | 0.4 | 0.1 | 11.01 | 0.04 |
| *G876* | 2,149 | 43 (34-54) | 0.00 | 12.5 | | 1,880 | | 0.3 | 0.1 | 10.42 | 0.03 |
| *G896* | 13,649 | 52 (39-76) | 0.02 | 24.9 | | 10,246 | | 10.2 | 0.1 | 1.75 | 0.18 |
| *G911* | 2,201 | 46 (36-69) | 0.01 | 27.0 | | 1,607 | | 0.4 | 0.1 | 6.51 | 0.02 |
| *G912* | 1,527 | 50 (40-67) | 0.00 | 6.1 | | 1,434 | | 1.4 | 0.1 | 1.83 | 0.03 |
| *G914* | 2,700 | 49.5 (38-70) | 0.00 | 24.8 | | 2,031 | | 0.3 | 0.1 | 9.52 | 0.03 |
| *G936* | 6,464 | 42 (35-54) | 0.01 | 31.8 | | 4,406 | | 1.2 | 0.1 | 5.19 | 0.06 |
| *G939* | 1,904 | 41 (34-53) | 0.00 | 15.9 | | 1,602 | | 0.5 | 0.1 | 4.86 | 0.02 |
| *G942* | 3,892 | 42 (35-54) | 0.01 | 29.2 | | 2,756 | | 0.5 | 0.1 | 8.76 | 0.04 |
| *G943* | 242,400 | 69 (48-76) | 0.13 | 25.2 | | 181,305 | | 96.8 | 49.5 | 3.93 | 3.80 |
| *G951* | 2,056 | 46 (38-58) | 0.01 | 13.4 | | 1,780 | | 0.3 | 0.1 | 9.90 | 0.03 |
| *G973* | 3,160 | 42 (34-52) | 0.00 | 20.7 | | 2,505 | | 0.5 | 0.1 | 7.57 | 0.04 |
| *G978* | 1,308,555 | 92 (65-101) | 0.35 | 80.4 | | 256,929 | | 98.7 | 70.4 | 6.98 | 6.88 |

**IID:** individual ID; **aln:** aligned; **read length:** median (1st quartile – 3rd quartile); **rmdup:** duplicates removed;

**cov:** coverage; **DP cov:** read depth over covered bases; **DP all:** read depth over complete reference.

Table 3: *M. leprae* reads from Malt analysis

| ***SID*** | ***#reads*** *M. leprae* | ***#reads total*** | *M. leprae* ***proportion [%]*** |
| --- | --- | --- | --- |
| *G022* | 6,874 | 6,992,436 | 0.098 |
| *G102* | 331 | 17,688,112 | 0.002 |
| *G104* | 888 | 16,762,492 | 0.005 |
| *G1042* | 45 | 25,953,583 | 0.000 |
| *G1044* | 430 | 11,939,954 | 0.004 |
| *G1049* | 75 | 19,490,520 | 0.000 |
| *G1065* | 88 | 18,989,844 | 0.000 |
| *G1083* | 26,795 | 12,823,578 | 0.209 |
| *G1137* | 7,397 | 9,491,196 | 0.078 |
| *G1149* | 16,639 | 8,543,002 | 0.195 |
| *G117* | 97 | 10,235,336 | 0.001 |
| *G118* | 2,891 | 12,907,976 | 0.022 |
| *G119* | 860 | 14,841,003 | 0.006 |
| *G120* | 38 | 18,132,253 | 0.000 |
| *G131* | 6,871 | 17,278,424 | 0.040 |
| *G140* | 48 | 16,450,945 | 0.000 |
| *G149* | 741 | 7,921,056 | 0.009 |
| *G154* | 140,683 | 9,564,519 | 1.471 |
| *G164* | 6,505 | 16,248,305 | 0.040 |
| *G165* | 5,852 | 19,328,959 | 0.030 |
| *G166* | 1,014 | 28,018,881 | 0.004 |
| *G189* | 209 | 36,179,899 | 0.001 |
| *G208* | 20,692 | 15,799,820 | 0.131 |
| *G21* | 719 | 6,964,677 | 0.010 |
| *G24* | 1,271 | 30,498,430 | 0.004 |
| *G255* | 341 | 13,384,857 | 0.003 |
| *G274* | 404 | 14,859,656 | 0.003 |
| *G28* | 71 | 47,701,300 | 0.000 |
| *G289* | 2,578 | 16,927,524 | 0.015 |
| *G300* | 89 | 15,645,987 | 0.001 |
| *G314* | 75 | 14,889,641 | 0.001 |
| *G33* | 5,697 | 8,313,032 | 0.069 |
| *G34* | 87,695 | 19,710,929 | 0.445 |
| *G348* | 2,719 | 16,754,756 | 0.016 |
| *G393* | 114 | 14,001,015 | 0.001 |
| *G397* | 764 | 12,425,806 | 0.006 |
| *G404* | 52,858 | 21,352,057 | 0.248 |
| *G417* | 60 | 44,527,684 | 0.000 |
| *G427* | 181,858 | 28,508,113 | 0.638 |
| *G43* | 695 | 17,074,447 | 0.004 |
| *G472* | 12,531 | 9,623,786 | 0.130 |
| *G48* | 27 | 21,187,364 | 0.000 |
| *G507* | 2,624,974 | 19,464,646 | 13.486 |
| *G533* | 424,828 | 28,125,125 | 1.510 |
| *G611* | 5,571 | 26,768,800 | 0.021 |
| *G658* | 10,150 | 81,916,162 | 0.012 |
| *G669* | 6,462 | 20,146,302 | 0.032 |
| *G708* | 34 | 15,488,225 | 0.000 |
| *G712* | 6 | 13,565,885 | 0.000 |
| *G722* | 78,211 | 24,755,930 | 0.316 |
| *G730* | 2,130 | 12,976,982 | 0.016 |
| *G738* | 218 | 36,353,460 | 0.001 |
| *G749* | 1,868,106 | 22,577,271 | 8.274 |
| *G750* | 131 | 16,027,731 | 0.001 |
| *G860* | 262 | 20,727,017 | 0.001 |
| *G864* | 59 | 18,458,498 | 0.000 |
| *G870* | 49 | 17,782,756 | 0.000 |
| *G876* | 13 | 16,529,456 | 0.000 |
| *G896* | 5,225 | 47,824,741 | 0.011 |
| *G911* | 56 | 12,122,972 | 0.000 |
| *G912* | 751 | 16,458,021 | 0.005 |
| *G914* | 196 | 52,374,396 | 0.000 |
| *G936* | 372 | 18,568,653 | 0.002 |
| *G939* | 146 | 14,134,206 | 0.001 |
| *G942* | 95 | 18,190,020 | 0.001 |
| *G943* | 29,633 | 25,978,356 | 0.114 |
| *G951* | 41 | 16,358,004 | 0.000 |
| *G973* | 56 | 16,256,715 | 0.000 |
| *G978* | 17,548 | 22,021,382 | 0.080 |

**SID:** sample ID.

Table 4: Raw data, pre-processing and QC of UDG datasets

| ***IID*** | ***# reads raw*** | ***# reads merged*** | ***QC proportion [%]*** | ***merged proportion [%]*** |
| --- | --- | --- | --- | --- |
| *G022* | 30,940,834 | 18,719,919 | 98.22 | 91.36 |
| *G102* | 137,752,212 | 78,346,278 | 97.64 | 82.31 |
| *G104* | 64,773,586 | 35,058,852 | 98.53 | 89.84 |
| *G1042* | 66,529,964 | 37,455,713 | 97.83 | 84.57 |
| *G1044* | 64,698,698 | 42,130,966 | 97.79 | 63.34 |
| *G1049* | 107,712,188 | 62,069,503 | 98.37 | 82.02 |
| *G1065* | 176,912,136 | 101,902,722 | 98.23 | 81.90 |
| *G1083* | 770,645,748 | 427,854,448 | 97.77 | 86.34 |
| *G1137* | 444,240,476 | 238,536,795 | 98.28 | 94.27 |
| *G1149* | 887,506,230 | 499,982,897 | 97.25 | 83.85 |
| *G117* | 43,310,544 | 25,937,330 | 98.01 | 75.81 |
| *G118* | 65,155,022 | 42,167,892 | 98.05 | 65.35 |
| *G119* | 227,050,610 | 132,024,094 | 97.89 | 80.91 |
| *G120* | 130,859,124 | 81,667,917 | 98.40 | 72.03 |
| *G131* | 49,267,746 | 29,316,317 | 98.07 | 88.92 |
| *G140* | 79,083,200 | 47,839,848 | 98.21 | 75.23 |
| *G149* | 85,213,296 | 51,532,146 | 98.36 | 91.33 |
| *G154* | 524,900,604 | 302,927,227 | 97.81 | 81.64 |
| *G164* | 80,225,662 | 44,899,250 | 98.24 | 86.03 |
| *G165* | 112,600,952 | 65,084,926 | 98.63 | 82.25 |
| *G166* | 92,582,562 | 51,453,626 | 97.78 | 85.96 |
| *G189* | 160,762,140 | 91,904,986 | 98.27 | 86.45 |
| *G208* | 607,542,372 | 342,750,214 | 97.67 | 84.22 |
| *G21* | 140,118,210 | 73,602,276 | 97.61 | 91.95 |
| *G24* | 139,875,166 | 87,972,862 | 98.53 | 69.16 |
| *G255* | 68,617,716 | 42,105,641 | 98.24 | 73.02 |
| *G274* | 49,850,574 | 29,889,767 | 98.26 | 74.41 |
| *G28* | 100,553,030 | 60,441,387 | 97.66 | 76.55 |
| *G289* | 49,726,866 | 29,313,354 | 97.94 | 89.34 |
| *G300* | 115,357,416 | 71,147,275 | 97.91 | 69.25 |
| *G314* | 121,117,564 | 70,473,087 | 98.34 | 80.17 |
| *G33* | 488,857,644 | 284,464,170 | 97.59 | 80.48 |
| *G34* | 799,544,686 | 430,921,244 | 97.54 | 89.23 |
| *G348* | 61,323,054 | 34,863,984 | 98.48 | 83.41 |
| *G393* | 180,728,072 | 99,459,731 | 97.49 | 86.35 |
| *G397* | 63,283,818 | 38,089,772 | 98.22 | 75.95 |
| *G404* | 493,618,248 | 286,726,388 | 98.06 | 92.32 |
| *G417* | 130,525,388 | 69,432,608 | 97.84 | 91.10 |
| *G427* | 760,562,012 | 472,194,157 | 98.04 | 89.46 |
| *G43* | 196,198,708 | 112,242,084 | 98.17 | 82.72 |
| *G472* | 579,200,126 | 345,256,592 | 98.35 | 91.80 |
| *G48* | 83,999,240 | 46,556,337 | 98.94 | 87.29 |
| *G507* | 617,672,020 | 360,109,310 | 98.31 | 93.26 |
| *G533* | 126,463,738 | 77,389,431 | 98.06 | 87.06 |
| *G658* | 1,148,892,432 | 624,163,521 | 98.53 | 92.83 |
| *G669* | 224,364,814 | 123,218,314 | 98.20 | 87.94 |
| *G708* | 143,142,880 | 78,603,465 | 97.66 | 87.05 |
| *G712* | 96,660,500 | 54,150,249 | 96.41 | 81.80 |
| *G722* | 859,339,596 | 458,475,384 | 98.13 | 93.67 |
| *G730* | 122,309,144 | 63,145,152 | 96.71 | 93.08 |
| *G738* | 144,795,996 | 91,838,989 | 98.36 | 66.44 |
| *G749* | 120,782,402 | 68,511,590 | 98.09 | 92.74 |
| *G750* | 41,475,780 | 21,524,070 | 97.23 | 92.97 |
| *G860* | 126,024,086 | 74,135,890 | 98.54 | 79.70 |
| *G864* | 69,116,130 | 38,025,739 | 98.06 | 87.25 |
| *G870* | 71,143,658 | 41,070,437 | 96.86 | 78.69 |
| *G876* | 123,654,436 | 72,242,271 | 98.04 | 78.96 |
| *G896* | 133,833,908 | 77,575,165 | 97.59 | 80.72 |
| *G911* | 62,019,916 | 38,500,056 | 97.88 | 71.46 |
| *G912* | 54,518,512 | 34,333,648 | 98.32 | 67.47 |
| *G914* | 118,118,308 | 67,433,796 | 97.87 | 83.25 |
| *G936* | 156,957,074 | 102,869,340 | 98.18 | 67.50 |
| *G939* | 100,032,586 | 61,932,630 | 97.95 | 72.83 |
| *G942* | 119,015,656 | 72,399,849 | 98.17 | 75.08 |
| *G943* | 340,850,762 | 189,639,267 | 98.29 | 85.70 |
| *G951* | 66,378,072 | 40,912,665 | 98.24 | 71.35 |
| *G973* | 163,751,118 | 96,735,567 | 98.04 | 76.88 |
| *G978* | 631,852,292 | 370,246,332 | 97.87 | 92.79 |

**IID:** individual ID.

Table 5: *M. leprae* damage patterns of UDG datasets

| ***IID*** | ***DMG 1st base 5' [%]*** | ***DMG 2nd base 5' [%]*** | ***DMG 3rd base 5' [%]*** | ***DMG 4th base 5' [%]*** | ***DMG 5th base 5' [%]*** | ***DMG 1st base 3' [%]*** | ***DMG 2nd base 3' [%]*** | ***DMG 3rd base 3' [%]*** | ***DMG 4th base 3' [%]*** | ***DMG 5th base 3' [%]*** |
| --- | --- | --- | --- | --- | --- | --- | --- | --- | --- | --- |
| *G022* | 0.56 | 0.14 | 0.08 | 0.09 | 0.12 | 0.48 | 0.15 | 0.20 | 0.09 | 0.12 |
| *G102* | 1.41 | 1.94 | 1.42 | 0.89 | 1.11 | 1.51 | 2.51 | 1.65 | 1.18 | 2.17 |
| *G104* | 1.39 | 1.63 | 1.22 | 0.45 | 0.40 | 0.86 | 0.91 | 2.02 | 0.00 | 0.88 |
| *G1042* | 1.81 | 2.53 | 0.92 | 1.95 | 1.77 | 1.21 | 4.16 | 1.27 | 1.00 | 2.85 |
| *G1044* | 2.56 | 2.33 | 2.27 | 2.13 | 2.50 | 2.27 | 0.00 | 0.00 | 0.00 | 0.00 |
| *G1049* | 5.93 | 1.87 | 1.36 | 1.79 | 0.90 | 3.91 | 3.81 | 3.55 | 1.00 | 0.93 |
| *G1065* | 1.67 | 5.96 | 3.21 | 4.25 | 3.73 | 3.27 | 3.01 | 4.24 | 4.29 | 3.39 |
| *G1083* | 0.22 | 0.05 | 0.31 | 0.05 | 0.17 | 0.31 | 0.10 | 0.14 | 0.13 | 0.04 |
| *G1137* | 0.41 | 0.34 | 0.32 | 0.33 | 0.25 | 0.49 | 0.37 | 0.28 | 0.31 | 0.30 |
| *G1149* | 0.33 | 0.11 | 0.08 | 0.08 | 0.08 | 0.26 | 0.13 | 0.06 | 0.14 | 0.08 |
| *G117* | 3.03 | 0.00 | 5.41 | 8.82 | 0.00 | 0.00 | 0.00 | 0.00 | 0.00 | 2.94 |
| *G118* | 0.00 | 0.44 | 0.78 | 1.81 | 0.83 | 0.85 | 0.85 | 0.00 | 0.00 | 1.33 |
| *G119* | 1.56 | 1.59 | 1.98 | 1.12 | 1.53 | 2.70 | 1.63 | 1.24 | 2.18 | 2.29 |
| *G120* | 0.77 | 0.00 | 0.00 | 2.31 | 2.01 | 0.00 | 2.94 | 1.05 | 2.13 | 2.04 |
| *G131* | 0.90 | 0.47 | 0.16 | 0.25 | 0.15 | 0.64 | 0.37 | 0.36 | 0.32 | 0.34 |
| *G140* | 1.82 | 2.51 | 1.56 | 3.45 | 1.61 | 0.42 | 3.46 | 1.67 | 2.26 | 1.69 |
| *G149* | 1.30 | 0.29 | 0.93 | 0.68 | 0.13 | 1.14 | 0.88 | 0.41 | 0.68 | 0.68 |
| *G154* | 0.24 | 0.07 | 0.06 | 0.04 | 0.04 | 0.21 | 0.06 | 0.06 | 0.08 | 0.05 |
| *G164* | 1.35 | 1.15 | 0.28 | 0.44 | 0.85 | 1.85 | 1.24 | 0.53 | 0.66 | 0.45 |
| *G165* | 0.65 | 0.91 | 0.20 | 0.86 | 0.20 | 1.21 | 0.23 | 0.41 | 0.44 | 0.21 |
| *G166* | 1.45 | 2.31 | 1.37 | 2.27 | 2.53 | 1.69 | 3.44 | 1.48 | 2.01 | 2.59 |
| *G189* | 0.98 | 0.13 | 0.07 | 0.04 | 0.09 | 0.43 | 0.18 | 0.09 | 0.13 | 0.05 |
| *G208* | 0.22 | 0.11 | 0.17 | 0.15 | 0.06 | 0.23 | 0.09 | 0.13 | 0.15 | 0.13 |
| *G21* | 1.36 | 0.52 | 0.70 | 0.00 | 1.52 | 1.09 | 1.45 | 0.23 | 0.82 | 2.41 |
| *G24* | 1.78 | 1.47 | 1.24 | 1.59 | 1.28 | 1.66 | 1.38 | 1.06 | 1.05 | 0.17 |
| *G255* | 1.64 | 0.00 | 0.00 | 0.00 | 0.00 | 1.85 | 1.35 | 1.59 | 1.85 | 5.26 |
| *G274* | 2.22 | 0.00 | 2.04 | 5.88 | 0.00 | 0.00 | 0.00 | 1.82 | 0.00 | 1.79 |
| *G28* | 2.48 | 2.72 | 1.85 | 2.75 | 1.31 | 1.42 | 2.30 | 2.01 | 1.85 | 2.27 |
| *G289* | 1.40 | 0.71 | 0.51 | 0.57 | 0.55 | 0.47 | 1.15 | 0.99 | 0.88 | 0.38 |
| *G300* | 1.88 | 3.44 | 3.19 | 1.90 | 2.15 | 3.44 | 3.52 | 2.32 | 2.66 | 2.71 |
| *G314* | 3.72 | 3.93 | 2.95 | 3.33 | 3.06 | 1.32 | 1.22 | 2.54 | 1.00 | 2.22 |
| *G33* | 0.53 | 0.17 | 0.32 | 0.65 | 0.10 | 0.68 | 0.72 | 0.31 | 0.05 | 0.42 |
| *G34* | 0.15 | 0.05 | 0.07 | 0.11 | 0.12 | 0.26 | 0.14 | 0.07 | 0.09 | 0.03 |
| *G348* | 0.93 | 0.00 | 1.19 | 2.20 | 0.94 | 1.01 | 1.52 | 1.79 | 1.03 | 0.98 |
| *G393* | 3.14 | 2.84 | 2.36 | 1.35 | 0.00 | 2.16 | 1.92 | 1.73 | 2.59 | 2.24 |
| *G397* | 1.14 | 2.22 | 1.86 | 2.70 | 0.97 | 1.96 | 1.19 | 1.85 | 0.99 | 1.69 |
| *G404* | 0.32 | 0.15 | 0.10 | 0.11 | 0.09 | 0.37 | 0.17 | 0.11 | 0.12 | 0.11 |
| *G417* | 2.37 | 2.51 | 2.05 | 1.93 | 1.70 | 2.89 | 2.58 | 1.90 | 1.85 | 1.52 |
| *G427* | 0.40 | 0.14 | 0.25 | 0.10 | 0.08 | 0.46 | 0.20 | 0.16 | 0.15 | 0.12 |
| *G43* | 2.27 | 1.31 | 1.31 | 1.54 | 0.57 | 1.90 | 2.65 | 0.72 | 1.62 | 1.88 |
| *G472* | 0.56 | 0.18 | 0.15 | 0.13 | 0.11 | 0.59 | 0.21 | 0.18 | 0.13 | 0.14 |
| *G48* | 3.00 | 3.34 | 2.30 | 1.88 | 2.39 | 3.95 | 1.87 | 3.00 | 3.40 | 3.49 |
| *G507* | 0.24 | 0.06 | 0.05 | 0.05 | 0.04 | 0.26 | 0.11 | 0.08 | 0.08 | 0.07 |
| *G533* | 0.41 | 0.10 | 0.04 | 0.06 | 0.06 | 0.36 | 0.15 | 0.13 | 0.12 | 0.09 |
| *G658* | 0.82 | 0.65 | 0.37 | 0.40 | 0.44 | 0.90 | 0.68 | 0.46 | 0.45 | 0.41 |
| *G669* | 0.69 | 0.26 | 0.52 | 0.35 | 0.45 | 1.04 | 0.46 | 0.62 | 0.39 | 0.41 |
| *G708* | 3.25 | 2.04 | 2.73 | 1.25 | 2.42 | 3.83 | 2.94 | 2.36 | 2.48 | 1.35 |
| *G712* | 1.56 | 0.93 | 0.78 | 1.37 | 0.89 | 1.62 | 1.39 | 0.76 | 1.56 | 1.49 |
| *G722* | 0.41 | 0.12 | 0.11 | 0.09 | 0.09 | 0.39 | 0.19 | 0.14 | 0.13 | 0.13 |
| *G730* | 2.09 | 1.03 | 1.25 | 1.42 | 0.72 | 2.26 | 1.05 | 1.49 | 1.19 | 1.25 |
| *G738* | 1.30 | 2.40 | 1.83 | 1.64 | 2.29 | 1.96 | 2.32 | 1.86 | 1.31 | 1.62 |
| *G749* | 0.21 | 0.07 | 0.08 | 0.06 | 0.04 | 0.30 | 0.15 | 0.11 | 0.11 | 0.09 |
| *G750* | 0.00 | 0.00 | 0.00 | 0.00 | 3.57 | 3.13 | 0.00 | 0.00 | 0.00 | 0.00 |
| *G860* | 4.05 | 2.87 | 4.02 | 1.36 | 2.55 | 5.63 | 5.26 | 4.24 | 5.71 | 3.73 |
| *G864* | 0.00 | 4.44 | 0.00 | 1.92 | 3.70 | 2.04 | 1.92 | 2.08 | 0.00 | 0.00 |
| *G870* | 2.78 | 1.82 | 1.05 | 3.57 | 2.27 | 0.98 | 2.08 | 2.04 | 2.47 | 0.00 |
| *G876* | 3.27 | 4.56 | 1.38 | 2.50 | 2.22 | 3.83 | 3.86 | 2.06 | 3.70 | 2.30 |
| *G896* | 1.29 | 1.60 | 0.82 | 1.03 | 1.11 | 1.42 | 1.88 | 0.80 | 1.38 | 1.32 |
| *G911* | 0.00 | 2.00 | 3.70 | 2.22 | 0.00 | 0.00 | 3.70 | 2.04 | 0.00 | 0.00 |
| *G912* | 7.14 | 0.00 | 0.00 | 0.00 | 0.00 | 0.00 | 11.11 | 0.00 | 0.00 | 0.00 |
| *G914* | 2.90 | 5.33 | 2.10 | 1.99 | 2.42 | 2.09 | 2.72 | 4.02 | 3.06 | 1.84 |
| *G936* | 2.38 | 2.05 | 0.66 | 0.76 | 0.69 | 1.06 | 1.31 | 2.93 | 0.72 | 1.37 |
| *G939* | 2.92 | 2.41 | 2.40 | 0.92 | 2.11 | 2.82 | 1.75 | 2.34 | 0.89 | 0.90 |
| *G942* | 0.99 | 2.03 | 1.79 | 0.99 | 2.42 | 2.93 | 3.65 | 1.90 | 0.00 | 1.83 |
| *G943* | 0.15 | 0.23 | 0.13 | 0.17 | 0.13 | 0.23 | 0.21 | 0.21 | 0.25 | 0.08 |
| *G951* | 5.05 | 2.06 | 1.85 | 3.92 | 2.86 | 1.05 | 4.00 | 2.26 | 1.89 | 1.30 |
| *G973* | 2.56 | 3.30 | 2.45 | 2.37 | 3.02 | 2.23 | 3.07 | 2.21 | 0.98 | 1.30 |
| *G978* | 0.68 | 0.52 | 0.54 | 0.42 | 0.44 | 0.81 | 0.58 | 0.47 | 0.48 | 0.40 |

Shown are the frequencies of cytosine to thymine mutations per position from the 5'-ends and the frequencies of guanine to adenine mutations per position from the 3'-ends. **IID:** individual ID; **DMG:** damage

Table 6: *M. leprae* damage patterns of non-UDG datasets

| ***IID*** | ***DMG 1st base 5' [%]*** | ***DMG 2nd base 5' [%]*** | ***DMG 3rd base 5' [%]*** | ***DMG 4th base 5' [%]*** | ***DMG 5th base 5' [%]*** | ***DMG 1st base 3' [%]*** | ***DMG 2nd base 3' [%]*** | ***DMG 3rd base 3' [%]*** | ***DMG 4th base 3' [%]*** | ***DMG 5th base 3' [%]*** |
| --- | --- | --- | --- | --- | --- | --- | --- | --- | --- | --- |
| *G022* | 3.685 | 3.360 | 1.367 | 0.653 | 0.553 | 3.096 | 3.243 | 1.061 | 0.708 | 0.310 |
| *G102* | 3.141 | 4.867 | 2.424 | 2.194 | 1.990 | 2.952 | 4.994 | 3.141 | 2.888 | 2.123 |
| *G104* | 7.222 | 4.801 | 2.682 | 1.925 | 1.808 | 6.685 | 4.790 | 3.781 | 2.174 | 2.430 |
| *G1042* | 6.915 | 4.020 | 3.704 | 2.235 | 4.023 | 6.857 | 8.284 | 4.918 | 4.615 | 4.420 |
| *G1044* | 9.697 | 6.164 | 3.419 | 2.570 | 4.328 | 7.520 | 7.237 | 3.191 | 2.954 | 3.695 |
| *G1049* | 11.047 | 2.362 | 4.861 | 5.056 | 2.395 | 5.650 | 5.682 | 4.268 | 5.660 | 4.110 |
| *G1065* | 8.654 | 6.030 | 5.025 | 4.846 | 2.210 | 10.288 | 5.348 | 3.721 | 3.211 | 4.639 |
| *G1083* | 8.998 | 8.086 | 3.235 | 2.036 | 1.580 | 8.987 | 8.188 | 3.262 | 2.238 | 1.462 |
| *G1137* | 3.326 | 2.566 | 1.364 | 0.981 | 1.169 | 2.817 | 3.004 | 1.290 | 0.986 | 0.689 |
| *G1149* | 6.724 | 6.452 | 3.052 | 2.186 | 1.291 | 6.700 | 6.417 | 3.041 | 2.239 | 1.423 |
| *G117* | 6.077 | 5.978 | 2.762 | 1.724 | 1.714 | 4.762 | 3.977 | 0.532 | 5.056 | 5.233 |
| *G118* | 7.182 | 5.825 | 2.739 | 1.564 | 1.519 | 7.197 | 5.040 | 2.149 | 1.954 | 1.348 |
| *G119* | 6.134 | 4.447 | 2.495 | 2.529 | 1.741 | 5.627 | 5.176 | 2.300 | 2.670 | 2.545 |
| *G120* | 5.350 | 2.740 | 4.724 | 2.844 | 2.174 | 6.931 | 6.122 | 2.811 | 2.899 | 2.252 |
| *G131* | 4.953 | 5.085 | 2.188 | 1.251 | 0.927 | 4.405 | 5.370 | 1.917 | 1.365 | 1.193 |
| *G140* | 11.111 | 8.173 | 4.835 | 5.155 | 4.656 | 9.957 | 7.551 | 5.192 | 5.814 | 3.712 |
| *G149* | 3.270 | 3.662 | 1.411 | 1.098 | 0.683 | 3.361 | 3.559 | 1.532 | 1.071 | 0.910 |
| *G154* | 5.068 | 3.774 | 1.825 | 1.459 | 1.140 | 4.842 | 3.877 | 1.846 | 1.323 | 1.140 |
| *G164* | 8.156 | 5.873 | 3.445 | 2.924 | 2.087 | 7.832 | 6.195 | 3.450 | 2.565 | 2.002 |
| *G165* | 6.684 | 4.873 | 2.706 | 1.482 | 1.722 | 5.434 | 5.311 | 3.050 | 2.575 | 1.870 |
| *G166* | 8.575 | 7.287 | 4.743 | 4.545 | 3.529 | 10.971 | 5.874 | 5.860 | 4.586 | 2.914 |
| *G189* | 3.543 | 3.521 | 1.327 | 0.928 | 0.695 | 3.376 | 3.649 | 1.351 | 1.027 | 0.734 |
| *G208* | 5.469 | 5.138 | 2.362 | 1.773 | 5.242 | 6.182 | 4.898 | 2.137 | 3.042 | 4.701 |
| *G21* | 7.592 | 6.051 | 2.116 | 2.383 | 1.339 | 5.753 | 5.710 | 3.033 | 1.561 | 1.235 |
| *G24* | 5.263 | 7.500 | 5.022 | 3.481 | 1.667 | 5.890 | 4.915 | 2.723 | 2.665 | 2.742 |
| *G255* | 4.872 | 3.311 | 4.190 | 3.303 | 1.582 | 4.651 | 4.950 | 2.695 | 3.170 | 1.412 |
| *G274* | 12.868 | 6.355 | 6.230 | 2.069 | 3.546 | 7.278 | 4.194 | 2.222 | 1.767 | 2.703 |
| *G28* | 6.452 | 7.663 | 2.048 | 2.439 | 2.083 | 5.435 | 5.674 | 2.482 | 3.929 | 2.105 |
| *G289* | 5.435 | 5.479 | 6.410 | 5.882 | 2.469 | 6.667 | 2.632 | 0.000 | 6.098 | 2.564 |
| *G300* | 3.409 | 4.290 | 1.878 | 0.957 | 0.897 | 3.384 | 4.843 | 1.372 | 1.021 | 0.642 |
| *G314* | 7.366 | 8.740 | 4.857 | 3.433 | 2.517 | 8.617 | 6.631 | 3.233 | 4.082 | 3.555 |
| *G33* | 3.448 | 6.250 | 4.615 | 3.947 | 2.500 | 6.607 | 5.351 | 3.323 | 3.072 | 1.899 |
| *G34* | 7.098 | 6.931 | 3.339 | 2.761 | 1.593 | 6.961 | 7.434 | 3.512 | 3.049 | 1.856 |
| *G348* | 5.987 | 4.984 | 1.933 | 1.324 | 0.936 | 5.695 | 4.583 | 1.771 | 1.270 | 0.931 |
| *G393* | 8.148 | 5.217 | 3.279 | 5.263 | 1.754 | 10.738 | 8.000 | 6.202 | 3.906 | 6.667 |
| *G397* | 7.457 | 5.271 | 3.380 | 2.378 | 2.476 | 6.061 | 4.090 | 3.889 | 3.605 | 2.219 |
| *G404* | 0.000 | 3.030 | 0.000 | 2.439 | 0.000 | 0.000 | 2.439 | 0.000 | 0.000 | 0.000 |
| *G417* | 8.840 | 8.023 | 4.816 | 4.457 | 4.533 | 6.793 | 9.440 | 5.385 | 5.615 | 3.692 |
| *G427* | 3.806 | 4.087 | 1.393 | 0.765 | 0.610 | 3.652 | 3.761 | 1.229 | 0.911 | 0.579 |
| *G43* | 6.473 | 4.404 | 1.980 | 2.331 | 2.743 | 4.440 | 5.275 | 3.474 | 2.804 | 1.587 |
| *G472* | 3.181 | 3.310 | 1.097 | 0.724 | 0.487 | 3.220 | 3.291 | 1.142 | 0.738 | 0.516 |
| *G48* | 5.769 | 4.196 | 2.941 | 4.651 | 3.378 | 6.863 | 4.545 | 4.324 | 4.785 | 2.454 |
| *G507* | 7.182 | 6.685 | 2.460 | 1.743 | 1.211 | 7.291 | 6.506 | 2.248 | 1.560 | 1.015 |
| *G533* | 4.681 | 4.987 | 1.630 | 1.024 | 0.727 | 4.859 | 4.908 | 1.710 | 1.079 | 0.751 |
| *G658* | 3.769 | 3.946 | 1.713 | 1.415 | 1.871 | 4.017 | 3.953 | 1.386 | 1.603 | 1.063 |
| *G669* | 5.894 | 6.809 | 4.697 | 2.558 | 1.784 | 6.563 | 7.558 | 4.019 | 2.372 | 2.021 |
| *G708* | 5.085 | 5.833 | 2.278 | 3.333 | 2.400 | 6.954 | 9.264 | 3.646 | 2.676 | 3.904 |
| *G712* | 7.184 | 7.640 | 6.138 | 5.413 | 5.433 | 7.225 | 6.408 | 6.383 | 4.977 | 3.814 |
| *G722* | 3.377 | 3.154 | 1.209 | 0.860 | 0.646 | 3.407 | 3.579 | 1.304 | 0.928 | 0.695 |
| *G730* | 5.106 | 5.497 | 4.006 | 2.450 | 2.428 | 5.180 | 5.582 | 3.809 | 2.735 | 2.163 |
| *G738* | 2.752 | 3.927 | 2.475 | 1.458 | 2.145 | 1.609 | 7.261 | 3.030 | 3.804 | 3.561 |
| *G749* | 4.278 | 4.599 | 1.564 | 0.930 | 0.582 | 4.171 | 4.482 | 1.567 | 0.884 | 0.617 |
| *G750* | 3.243 | 3.659 | 4.571 | 3.226 | 1.852 | 4.545 | 6.040 | 1.163 | 1.170 | 2.410 |
| *G860* | 11.712 | 4.843 | 5.250 | 4.071 | 5.674 | 7.416 | 6.767 | 6.863 | 3.571 | 3.947 |
| *G864* | 6.803 | 2.963 | 2.857 | 5.926 | 5.085 | 10.625 | 4.412 | 5.556 | 5.960 | 5.109 |
| *G870* | 8.428 | 4.595 | 4.296 | 4.487 | 4.630 | 6.430 | 5.926 | 2.592 | 4.009 | 2.331 |
| *G876* | 5.460 | 5.993 | 1.840 | 2.167 | 1.701 | 3.416 | 6.478 | 2.647 | 2.000 | 1.068 |
| *G896* | 7.558 | 5.189 | 2.673 | 2.548 | 2.336 | 8.121 | 5.482 | 3.759 | 2.219 | 2.950 |
| *G911* | 2.806 | 3.911 | 1.667 | 1.289 | 2.732 | 3.684 | 3.958 | 3.535 | 3.684 | 4.393 |
| *G912* | 6.489 | 5.941 | 4.396 | 1.916 | 3.600 | 4.029 | 4.215 | 4.498 | 3.584 | 3.019 |
| *G914* | 3.788 | 2.586 | 4.878 | 1.449 | 3.478 | 4.348 | 4.032 | 4.545 | 3.008 | 0.847 |
| *G936* | 7.239 | 6.271 | 5.325 | 2.228 | 3.333 | 8.101 | 3.630 | 3.416 | 3.966 | 2.215 |
| *G939* | 7.759 | 5.941 | 6.612 | 0.000 | 7.368 | 8.197 | 6.000 | 2.703 | 0.855 | 5.882 |
| *G942* | 7.430 | 8.664 | 5.523 | 4.673 | 4.965 | 5.431 | 6.646 | 3.324 | 2.500 | 1.899 |
| *G943* | 3.639 | 2.699 | 1.900 | 1.440 | 1.353 | 3.482 | 3.018 | 1.449 | 1.639 | 1.123 |
| *G951* | 4.317 | 5.067 | 2.703 | 3.788 | 2.663 | 4.478 | 6.000 | 5.277 | 2.145 | 3.958 |
| *G973* | 8.025 | 2.414 | 5.921 | 4.310 | 2.847 | 5.028 | 7.904 | 6.291 | 2.367 | 1.087 |
| *G978* | 3.482 | 3.564 | 1.045 | 0.737 | 0.645 | 3.257 | 3.387 | 1.425 | 0.678 | 0.465 |

Shown are the frequencies of cytosine to thymine mutations per position from the 5'-ends and the frequencies of guanine to adenine mutations per position from the 3'-ends. **IID:** individual ID; **DMG:** damage.**Table 7: *H. sapiens* alignments**

| ***IID*** | ***# reads aligned*** | ***read length*** | ***endogenous***  ***DNA [%]*** | ***duplicate***  ***proportion [%]*** | ***# reads, duplicates***  ***removed*** | ***rmdup cov >= 1x [%]*** | ***rmdup  cov >= 4x [%]*** | ***DP***  ***cov*** | ***DP***  ***all*** |
| --- | --- | --- | --- | --- | --- | --- | --- | --- | --- |
| *G022* | 215,766 | 77 (57-101) | 1.2 | 21.4 | 169,618 | 0.40 | 0.00 | 1.06 | 0.00 |
| *G102* | 23,579,580 | 54 (42-72) | 30.1 | 26.5 | 17,334,404 | 18.51 | 1.08 | 1.62 | 0.30 |
| *G104* | 6,605,438 | 51 (41-66) | 18.8 | 22.8 | 5,102,603 | 6.92 | 0.09 | 1.22 | 0.08 |
| *G1042* | 701,195 | 47 (39-61) | 1.9 | 33.6 | 465,462 | 0.62 | 0.01 | 1.14 | 0.01 |
| *G1044* | 13,622,623 | 75 (61-76) | 32.3 | 51.3 | 6,630,800 | 8.32 | 0.69 | 1.74 | 0.14 |
| *G1049* | 23,481,725 | 61 (47-76) | 37.8 | 35.8 | 15,067,095 | 15.38 | 1.13 | 1.83 | 0.28 |
| *G1065* | 67,645,323 | 62 (45-76) | 66.4 | 17.7 | 55,684,519 | 44.95 | 6.10 | 2.41 | 1.08 |
| *G1083* | 19,687,515 | 67 (48-76) | 4.6 | 25.6 | 14,641,297 | 23.76 | 0.19 | 1.27 | 0.30 |
| *G1137* | 23,228,448 | 84 (61-109) | 9.7 | 48.9 | 11,859,028 | 24.40 | 0.36 | 1.35 | 0.33 |
| *G1149* | 109,397,923 | 68 (48-76) | 21.9 | 19.9 | 87,660,017 | 67.90 | 12.12 | 2.67 | 1.81 |
| *G117* | 1,124,488 | 68 (50-76) | 4.3 | 47.7 | 587,917 | 0.81 | 0.02 | 1.40 | 0.01 |
| *G118* | 1,126,737 | 74 (54-76) | 2.7 | 39.0 | 687,234 | 1.16 | 0.01 | 1.24 | 0.01 |
| *G119* | 35,280,597 | 58 (46-75) | 26.7 | 48.7 | 18,085,956 | 13.96 | 1.64 | 2.31 | 0.32 |
| *G120* | 31,932,003 | 55 (44-71) | 39.1 | 34.0 | 21,066,283 | 17.89 | 1.76 | 2.05 | 0.37 |
| *G131* | 430,351 | 76 (55-101) | 1.5 | 13.8 | 370,943 | 0.88 | 0.00 | 1.05 | 0.01 |
| *G140* | 5,279,932 | 64 (48-76) | 11.0 | 40.5 | 3,139,567 | 4.06 | 0.14 | 1.47 | 0.06 |
| *G149* | 7,448,353 | 87 (66-107) | 14.5 | 66.4 | 2,502,967 | 5.63 | 0.07 | 1.25 | 0.07 |
| *G154* | 126,215,593 | 68 (49-76) | 41.7 | 32.3 | 85,401,727 | 73.36 | 13.23 | 2.43 | 1.78 |
| *G164* | 1,826,283 | 67 (49-76) | 4.1 | 32.2 | 1,237,956 | 2.16 | 0.02 | 1.16 | 0.03 |
| *G165* | 27,121,754 | 62 (48-76) | 41.7 | 40.7 | 16,077,731 | 15.02 | 1.43 | 2.02 | 0.30 |
| *G166* | 2,458,635 | 76 (56-77) | 4.8 | 57.4 | 1,047,607 | 2.04 | 0.01 | 1.09 | 0.02 |
| *G189* | 19,398,801 | 74 (55-94) | 21.1 | 43.6 | 10,944,833 | 18.97 | 0.36 | 1.34 | 0.25 |
| *G208* | 107,762,330 | 60 (45-76) | 31.4 | 18.7 | 87,608,468 | 67.89 | 11.40 | 2.48 | 1.69 |
| *G21* | 48,361,116 | 48 (38-62) | 65.7 | 22.1 | 37,649,723 | 32.30 | 2.36 | 1.84 | 0.60 |
| *G24* | 23,152,458 | 55 (44-69) | 26.3 | 9.3 | 21,005,863 | 24.47 | 0.87 | 1.53 | 0.38 |
| *G255* | 4,057,061 | 73 (54-76) | 9.6 | 51.5 | 1,969,444 | 2.54 | 0.08 | 1.55 | 0.04 |
| *G274* | 1,505,461 | 71 (51-76) | 5.0 | 34.3 | 988,664 | 1.64 | 0.02 | 1.23 | 0.02 |
| *G28* | 7,415,638 | 76 (59-78) | 12.3 | 41.6 | 4,327,130 | 8.46 | 0.04 | 1.13 | 0.10 |
| *G289* | 405,863 | 85 (61-113) | 1.4 | 12.2 | 356,382 | 0.93 | 0.00 | 1.06 | 0.01 |
| *G300* | 30,649,672 | 59 (44-76) | 43.1 | 10.7 | 27,356,603 | 30.48 | 1.68 | 1.69 | 0.52 |
| *G314* | 38,748,493 | 60 (45-76) | 55.0 | 16.2 | 32,489,459 | 33.38 | 2.60 | 1.85 | 0.62 |
| *G33* | 27,530,338 | 58 (43-76) | 9.7 | 17.1 | 22,814,256 | 28.72 | 0.98 | 1.49 | 0.43 |
| *G34* | 157,386,794 | 54 (41-74) | 36.5 | 11.3 | 139,603,058 | 81.10 | 26.14 | 3.13 | 2.53 |
| *G348* | 6,470,911 | 70 (49-76) | 18.6 | 24.4 | 4,894,059 | 8.17 | 0.13 | 1.25 | 0.10 |
| *G393* | 48,039,012 | 49 (38-65) | 48.3 | 24.0 | 36,530,592 | 30.18 | 2.51 | 1.90 | 0.57 |
| *G397* | 5,969,861 | 75 (60-76) | 15.7 | 49.1 | 3,037,770 | 4.54 | 0.16 | 1.46 | 0.07 |
| *G404* | 25,385,167 | 85 (61-107) | 8.9 | 30.4 | 17,658,726 | 32.54 | 1.00 | 1.47 | 0.48 |
| *G417* | 10,482,705 | 75 (52-77) | 15.1 | 50.0 | 5,238,362 | 9.08 | 0.08 | 1.18 | 0.11 |
| *G427* | 21,881,009 | 91 (64-109) | 4.6 | 42.6 | 12,567,597 | 26.10 | 0.45 | 1.34 | 0.35 |
| *G43* | 68,786,622 | 62 (46-76) | 61.3 | 23.5 | 52,600,160 | 43.27 | 5.69 | 2.38 | 1.03 |
| *G472* | 50,728,779 | 74 (56-96) | 14.7 | 47.9 | 26,446,750 | 39.22 | 1.69 | 1.61 | 0.63 |
| *G48* | 23,566,885 | 55 (44-71) | 50.6 | 13.5 | 20,395,812 | 25.32 | 0.76 | 1.46 | 0.37 |
| *G507* | 156,000,511 | 80 (58-102) | 43.3 | 40.5 | 92,778,696 | 77.12 | 24.49 | 3.10 | 2.39 |
| *G533* | 13,130,183 | 93 (68-123) | 17.0 | 41.4 | 7,691,397 | 17.68 | 0.20 | 1.26 | 0.22 |
| *G658* | 53,805,598 | 78 (59-107) | 8.6 | 21.6 | 42,180,864 | 59.43 | 4.42 | 1.87 | 1.11 |
| *G669* | 74,989,553 | 57 (42-75) | 60.9 | 23.0 | 57,763,833 | 44.44 | 5.62 | 2.36 | 1.05 |
| *G708* | 24,763,743 | 55 (43-71) | 31.5 | 25.6 | 18,436,347 | 20.41 | 1.07 | 1.58 | 0.32 |
| *G712* | 18,369,162 | 59 (44-76) | 33.9 | 12.9 | 16,000,054 | 18.22 | 0.92 | 1.62 | 0.30 |
| *G722* | 66,654,787 | 82 (61-112) | 14.5 | 28.7 | 47,526,461 | 65.66 | 5.94 | 1.98 | 1.30 |
| *G730* | 25,385,975 | 50 (39-65) | 40.2 | 23.3 | 19,477,058 | 19.28 | 1.02 | 1.64 | 0.32 |
| *G738* | 4,158,513 | 64 (48-76) | 4.5 | 14.9 | 3,540,589 | 6.18 | 0.05 | 1.17 | 0.07 |
| *G749* | 21,350,469 | 97 (67-125) | 31.2 | 50.8 | 10,513,514 | 23.54 | 0.28 | 1.31 | 0.31 |
| *G750* | 2,145,646 | 53 (41-71) | 10.0 | 12.7 | 1,873,731 | 2.82 | 0.03 | 1.16 | 0.03 |
| *G860* | 52,503,851 | 63 (49-76) | 70.8 | 20.2 | 41,906,970 | 35.71 | 4.65 | 2.32 | 0.83 |
| *G864* | 26,626,110 | 58 (43-75) | 70.0 | 14.4 | 22,802,010 | 24.91 | 1.38 | 1.70 | 0.42 |
| *G870* | 6,025,073 | 66 (48-76) | 14.7 | 43.5 | 3,401,596 | 4.38 | 0.17 | 1.48 | 0.06 |
| *G876* | 36,938,203 | 60 (45-76) | 51.1 | 12.6 | 32,293,745 | 31.87 | 2.64 | 1.92 | 0.61 |
| *G896* | 22,595,083 | 76 (66-80) | 29.1 | 45.6 | 12,294,011 | 21.22 | 0.48 | 1.33 | 0.28 |
| *G911* | 17,959,426 | 76 (62-78) | 46.6 | 49.3 | 9,112,255 | 13.36 | 0.79 | 1.56 | 0.21 |
| *G912* | 6,981,697 | 73 (53-76) | 20.3 | 37.6 | 4,360,023 | 6.21 | 0.24 | 1.44 | 0.09 |
| *G914* | 9,197,212 | 61 (48-76) | 13.6 | 31.3 | 6,322,908 | 10.09 | 0.09 | 1.19 | 0.12 |
| *G936* | 31,887,982 | 58 (46-75) | 31.0 | 42.7 | 18,281,076 | 12.90 | 2.05 | 2.52 | 0.33 |
| *G939* | 31,101,522 | 74 (52-76) | 50.2 | 42.4 | 17,909,657 | 21.91 | 1.67 | 1.71 | 0.38 |
| *G942* | 25,149,579 | 75 (62-76) | 34.7 | 55.2 | 11,264,001 | 13.92 | 1.20 | 1.79 | 0.25 |
| *G943* | 2,528,822 | 55 (43-76) | 1.3 | 19.6 | 2,033,321 | 3.34 | 0.01 | 1.12 | 0.04 |
| *G951* | 4,408,246 | 61 (48-76) | 10.8 | 18.1 | 3,610,547 | 5.36 | 0.10 | 1.29 | 0.07 |
| *G973* | 49,117,138 | 66 (48-76) | 50.8 | 23.2 | 37,719,032 | 37.08 | 3.79 | 2.05 | 0.76 |
| *G978* | 9,591,242 | 101 (73-115) | 2.6 | 46.8 | 5,098,219 | 12.70 | 0.09 | 1.20 | 0.15 |

**IID:** individual ID; **aln:** aligned; read length: median (1st quartile – 3rd quartile); **rmdup:** duplicates removed;

**cov:** coverage; **DP cov:** read depth over covered bases; **DP all:** read depth over complete reference.

Table 8: *H. sapiens* damage patterns of UDG datasets

| ***IID*** | ***DMG 1st base 5' [%]*** | ***DMG 2nd base 5' [%]*** | ***DMG 3rd base 5' [%]*** | ***DMG 4th base 5' [%]*** | ***DMG 5th base 5' [%]*** | ***DMG 1st base 3' [%]*** | ***DMG 2nd base 3' [%]*** | ***DMG 3rd base 3' [%]*** | ***DMG 4th base 3' [%]*** | ***DMG 5th base 3' [%]*** |
| --- | --- | --- | --- | --- | --- | --- | --- | --- | --- | --- |
| *G022* | 1.49 | 0.74 | 0.51 | 0.43 | 0.36 | 1.49 | 0.86 | 0.63 | 0.51 | 0.45 |
| *G102* | 1.49 | 0.81 | 0.57 | 0.42 | 0.35 | 1.54 | 0.82 | 0.56 | 0.43 | 0.34 |
| *G104* | 1.57 | 0.85 | 0.59 | 0.44 | 0.38 | 1.51 | 0.82 | 0.58 | 0.44 | 0.39 |
| *G1042* | 0.97 | 0.82 | 0.54 | 0.42 | 0.43 | 0.92 | 0.84 | 0.54 | 0.49 | 0.41 |
| *G1044* | 1.25 | 0.70 | 0.52 | 0.37 | 0.35 | 1.17 | 0.68 | 0.46 | 0.37 | 0.33 |
| *G1049* | 1.69 | 1.05 | 0.82 | 0.64 | 0.56 | 1.55 | 0.97 | 0.77 | 0.62 | 0.57 |
| *G1065* | 1.39 | 0.79 | 0.61 | 0.48 | 0.40 | 1.45 | 0.79 | 0.61 | 0.48 | 0.40 |
| *G1083* | 1.18 | 0.59 | 0.51 | 0.36 | 0.32 | 1.09 | 0.58 | 0.45 | 0.35 | 0.33 |
| *G1137* | 1.28 | 0.73 | 0.48 | 0.38 | 0.32 | 1.35 | 0.76 | 0.49 | 0.40 | 0.34 |
| *G1149* | 1.68 | 0.91 | 0.69 | 0.56 | 0.44 | 1.74 | 0.91 | 0.69 | 0.56 | 0.46 |
| *G117* | 0.94 | 0.48 | 0.41 | 0.30 | 0.33 | 0.83 | 0.48 | 0.35 | 0.31 | 0.24 |
| *G118* | 0.91 | 0.59 | 0.45 | 0.37 | 0.31 | 0.85 | 0.51 | 0.42 | 0.34 | 0.26 |
| *G119* | 1.01 | 0.68 | 0.60 | 0.45 | 0.41 | 0.93 | 0.63 | 0.57 | 0.43 | 0.39 |
| *G120* | 1.40 | 0.87 | 0.68 | 0.47 | 0.43 | 1.29 | 0.82 | 0.63 | 0.45 | 0.42 |
| *G131* | 1.74 | 0.69 | 0.49 | 0.42 | 0.38 | 1.61 | 0.77 | 0.55 | 0.44 | 0.39 |
| *G140* | 1.32 | 0.83 | 0.64 | 0.43 | 0.40 | 1.16 | 0.74 | 0.55 | 0.45 | 0.37 |
| *G149* | 1.30 | 0.65 | 0.46 | 0.35 | 0.32 | 1.40 | 0.69 | 0.50 | 0.40 | 0.34 |
| *G154* | 1.73 | 0.91 | 0.65 | 0.53 | 0.44 | 1.65 | 0.85 | 0.63 | 0.51 | 0.42 |
| *G164* | 1.03 | 0.61 | 0.54 | 0.38 | 0.31 | 1.01 | 0.59 | 0.45 | 0.37 | 0.33 |
| *G165* | 1.09 | 0.87 | 0.66 | 0.50 | 0.44 | 1.02 | 0.80 | 0.62 | 0.46 | 0.43 |
| *G166* | 1.03 | 1.07 | 0.50 | 0.47 | 0.37 | 0.99 | 1.05 | 0.49 | 0.50 | 0.39 |
| *G189* | 1.97 | 0.67 | 0.43 | 0.33 | 0.27 | 1.57 | 0.74 | 0.47 | 0.36 | 0.30 |
| *G208* | 1.51 | 0.78 | 0.56 | 0.45 | 0.36 | 1.56 | 0.78 | 0.55 | 0.44 | 0.35 |
| *G21* | 1.83 | 1.04 | 0.79 | 0.64 | 0.51 | 1.90 | 1.04 | 0.79 | 0.63 | 0.50 |
| *G24* | 1.54 | 0.91 | 0.66 | 0.53 | 0.46 | 1.48 | 0.91 | 0.67 | 0.53 | 0.47 |
| *G255* | 1.17 | 0.67 | 0.45 | 0.34 | 0.32 | 0.99 | 0.62 | 0.44 | 0.33 | 0.29 |
| *G274* | 1.23 | 0.67 | 0.56 | 0.37 | 0.31 | 1.20 | 0.58 | 0.42 | 0.35 | 0.28 |
| *G28* | 0.85 | 0.92 | 0.46 | 0.42 | 0.32 | 0.88 | 0.88 | 0.45 | 0.41 | 0.32 |
| *G289* | 1.28 | 0.66 | 0.42 | 0.32 | 0.30 | 1.17 | 0.70 | 0.44 | 0.39 | 0.36 |
| *G300* | 1.59 | 0.74 | 0.58 | 0.47 | 0.38 | 1.63 | 0.76 | 0.58 | 0.47 | 0.37 |
| *G314* | 1.56 | 0.82 | 0.59 | 0.48 | 0.39 | 1.60 | 0.83 | 0.60 | 0.48 | 0.38 |
| *G33* | 1.60 | 0.90 | 0.67 | 0.54 | 0.43 | 1.64 | 0.91 | 0.67 | 0.53 | 0.42 |
| *G34* | 1.76 | 0.91 | 0.61 | 0.49 | 0.40 | 1.79 | 0.91 | 0.60 | 0.48 | 0.39 |
| *G348* | 1.21 | 0.75 | 0.50 | 0.41 | 0.34 | 1.15 | 0.69 | 0.45 | 0.41 | 0.35 |
| *G393* | 1.48 | 0.80 | 0.54 | 0.41 | 0.33 | 1.51 | 0.80 | 0.54 | 0.41 | 0.32 |
| *G397* | 1.13 | 0.70 | 0.50 | 0.35 | 0.33 | 1.08 | 0.57 | 0.46 | 0.34 | 0.31 |
| *G404* | 2.57 | 1.18 | 0.78 | 0.62 | 0.51 | 2.63 | 1.20 | 0.80 | 0.64 | 0.52 |
| *G417* | 1.11 | 0.67 | 0.41 | 0.33 | 0.27 | 1.10 | 0.66 | 0.41 | 0.33 | 0.28 |
| *G427* | 1.84 | 0.86 | 0.59 | 0.44 | 0.37 | 1.85 | 0.90 | 0.58 | 0.46 | 0.37 |
| *G43* | 1.32 | 0.69 | 0.54 | 0.43 | 0.36 | 1.35 | 0.70 | 0.54 | 0.43 | 0.36 |
| *G472* | 1.37 | 0.68 | 0.51 | 0.39 | 0.33 | 1.41 | 0.72 | 0.54 | 0.42 | 0.36 |
| *G48* | 1.07 | 0.71 | 0.52 | 0.41 | 0.35 | 1.07 | 0.69 | 0.50 | 0.40 | 0.35 |
| *G507* | 2.06 | 0.93 | 0.61 | 0.49 | 0.39 | 2.12 | 0.98 | 0.63 | 0.51 | 0.41 |
| *G533* | 1.95 | 0.93 | 0.61 | 0.52 | 0.41 | 1.85 | 0.97 | 0.66 | 0.56 | 0.45 |
| *G658* | 0.90 | 0.55 | 0.36 | 0.31 | 0.27 | 0.97 | 0.58 | 0.40 | 0.32 | 0.28 |
| *G669* | 0.92 | 0.73 | 0.45 | 0.45 | 0.37 | 0.96 | 0.74 | 0.44 | 0.44 | 0.37 |
| *G708* | 1.79 | 0.95 | 0.64 | 0.49 | 0.38 | 1.79 | 0.94 | 0.62 | 0.49 | 0.38 |
| *G712* | 1.53 | 1.01 | 0.72 | 0.60 | 0.52 | 1.59 | 1.01 | 0.74 | 0.61 | 0.52 |
| *G722* | 1.07 | 0.50 | 0.37 | 0.28 | 0.24 | 1.05 | 0.56 | 0.40 | 0.32 | 0.27 |
| *G730* | 1.97 | 1.10 | 0.83 | 0.67 | 0.53 | 1.99 | 1.10 | 0.83 | 0.66 | 0.52 |
| *G738* | 1.18 | 0.77 | 0.53 | 0.43 | 0.34 | 1.15 | 0.72 | 0.55 | 0.44 | 0.37 |
| *G749* | 1.30 | 0.66 | 0.50 | 0.40 | 0.34 | 1.44 | 0.75 | 0.53 | 0.44 | 0.37 |
| *G750* | 1.11 | 0.80 | 0.53 | 0.45 | 0.37 | 0.99 | 0.72 | 0.49 | 0.38 | 0.37 |
| *G860* | 1.64 | 1.03 | 0.78 | 0.65 | 0.59 | 1.50 | 0.97 | 0.75 | 0.62 | 0.58 |
| *G864* | 1.22 | 0.78 | 0.58 | 0.49 | 0.42 | 1.17 | 0.75 | 0.57 | 0.47 | 0.41 |
| *G870* | 1.10 | 0.63 | 0.49 | 0.35 | 0.31 | 1.00 | 0.58 | 0.43 | 0.31 | 0.30 |
| *G876* | 1.41 | 0.89 | 0.70 | 0.55 | 0.47 | 1.45 | 0.89 | 0.68 | 0.55 | 0.47 |
| *G896* | 0.80 | 0.55 | 0.37 | 0.30 | 0.26 | 0.75 | 0.55 | 0.37 | 0.29 | 0.26 |
| *G911* | 1.36 | 0.69 | 0.55 | 0.38 | 0.32 | 1.25 | 0.67 | 0.48 | 0.36 | 0.31 |
| *G912* | 1.18 | 0.67 | 0.62 | 0.40 | 0.36 | 1.04 | 0.63 | 0.56 | 0.37 | 0.35 |
| *G914* | 1.06 | 1.17 | 0.66 | 0.58 | 0.49 | 1.05 | 1.16 | 0.67 | 0.59 | 0.50 |
| *G936* | 1.09 | 0.70 | 0.65 | 0.48 | 0.42 | 0.97 | 0.64 | 0.61 | 0.45 | 0.42 |
| *G939* | 0.98 | 0.55 | 0.38 | 0.29 | 0.24 | 0.95 | 0.54 | 0.37 | 0.29 | 0.24 |
| *G942* | 1.18 | 0.66 | 0.49 | 0.36 | 0.30 | 1.12 | 0.64 | 0.44 | 0.34 | 0.30 |
| *G943* | 1.63 | 0.88 | 0.72 | 0.55 | 0.45 | 1.60 | 0.96 | 0.65 | 0.51 | 0.45 |
| *G951* | 1.38 | 0.72 | 0.61 | 0.41 | 0.37 | 1.21 | 0.65 | 0.57 | 0.42 | 0.37 |
| *G973* | 1.40 | 0.69 | 0.49 | 0.37 | 0.30 | 1.46 | 0.69 | 0.47 | 0.38 | 0.31 |
| *G978* | 1.25 | 0.67 | 0.48 | 0.38 | 0.33 | 1.35 | 0.70 | 0.50 | 0.41 | 0.35 |

Shown are the frequencies of cytosine to thymine mutations per position from the 5'-ends and the frequencies of guanine to adenine mutations per position from the 3'-ends. **IID:** individual ID; **DMG:** damage

Table 9: *H. sapiens* damage patterns of non-UDG datasets

| ***IID*** | ***DMG 1st base 5' [%]*** | ***DMG 2nd base 5' [%]*** | ***DMG 3rd base 5' [%]*** | ***DMG 4th base 5' [%]*** | ***DMG 5th base 5' [%]*** | ***DMG 1st base 3' [%]*** | ***DMG 2nd base 3' [%]*** | ***DMG 3rd base 3' [%]*** | ***DMG 4th base 3' [%]*** | ***DMG 5th base 3' [%]*** |
| --- | --- | --- | --- | --- | --- | --- | --- | --- | --- | --- |
| *G022* | 7.02 | 5.15 | 3.62 | 2.96 | 2.49 | 6.85 | 4.94 | 3.40 | 2.92 | 2.41 |
| *G102* | 17.77 | 10.80 | 8.14 | 6.93 | 5.57 | 17.67 | 10.65 | 8.01 | 6.81 | 5.48 |
| *G104* | 17.39 | 9.84 | 7.66 | 6.48 | 5.51 | 17.41 | 9.81 | 7.54 | 6.42 | 5.49 |
| *G1042* | 19.59 | 12.60 | 10.80 | 10.04 | 8.72 | 19.73 | 13.04 | 10.47 | 10.10 | 9.05 |
| *G1044* | 10.92 | 6.72 | 4.63 | 3.91 | 3.31 | 10.91 | 6.59 | 4.56 | 3.83 | 3.32 |
| *G1049* | 18.12 | 10.81 | 8.68 | 7.82 | 7.14 | 18.28 | 10.67 | 8.57 | 7.83 | 7.09 |
| *G1065* | 13.54 | 8.20 | 6.38 | 5.63 | 4.58 | 13.58 | 8.16 | 6.26 | 5.53 | 4.50 |
| *G1083* | 11.40 | 6.78 | 4.92 | 4.13 | 3.54 | 11.34 | 6.80 | 4.89 | 4.07 | 3.49 |
| *G1137* | 7.28 | 4.92 | 3.30 | 2.77 | 2.35 | 7.38 | 5.11 | 3.36 | 2.81 | 2.41 |
| *G1149* | 19.07 | 13.68 | 10.63 | 9.05 | 6.71 | 18.96 | 13.42 | 10.41 | 8.90 | 6.58 |
| *G117* | 15.80 | 9.32 | 7.44 | 6.24 | 5.10 | 16.16 | 9.41 | 7.25 | 5.90 | 5.01 |
| *G118* | 9.38 | 5.89 | 4.25 | 3.49 | 2.99 | 9.54 | 5.68 | 4.15 | 3.45 | 2.99 |
| *G119* | 12.26 | 7.69 | 6.57 | 6.21 | 5.41 | 11.97 | 7.43 | 6.35 | 6.03 | 5.22 |
| *G120* | 20.72 | 11.36 | 9.27 | 8.32 | 7.37 | 20.84 | 11.36 | 9.21 | 8.32 | 7.35 |
| *G131* | 8.03 | 5.44 | 3.97 | 3.45 | 2.89 | 8.17 | 5.41 | 3.81 | 3.39 | 2.85 |
| *G140* | 15.22 | 9.05 | 7.20 | 6.19 | 5.42 | 15.60 | 8.95 | 7.19 | 6.19 | 5.56 |
| *G149* | 8.42 | 4.92 | 3.23 | 2.59 | 2.13 | 8.71 | 5.13 | 3.30 | 2.67 | 2.12 |
| *G154* | 17.64 | 10.57 | 7.93 | 6.82 | 5.90 | 17.65 | 10.49 | 7.76 | 6.77 | 5.81 |
| *G164* | 10.89 | 6.77 | 5.30 | 4.50 | 3.96 | 11.13 | 6.90 | 5.25 | 4.57 | 3.99 |
| *G165* | 13.41 | 7.67 | 6.33 | 5.71 | 5.08 | 13.49 | 7.71 | 6.41 | 5.73 | 5.07 |
| *G166* | 16.92 | 10.17 | 7.98 | 6.81 | 6.05 | 16.97 | 9.88 | 7.66 | 6.90 | 6.02 |
| *G189* | 8.72 | 5.22 | 3.40 | 2.75 | 2.13 | 8.65 | 5.11 | 3.31 | 2.72 | 2.14 |
| *G208* | 9.82 | 5.36 | 3.83 | 3.28 | 2.85 | 9.72 | 5.26 | 3.71 | 3.24 | 2.80 |
| *G21* | 16.25 | 10.34 | 7.19 | 6.04 | 4.75 | 16.13 | 10.15 | 7.04 | 5.97 | 4.65 |
| *G24* | 7.41 | 1.64 | 1.08 | 0.95 | 0.72 | 7.32 | 1.64 | 1.08 | 0.94 | 0.72 |
| *G255* | 14.25 | 8.02 | 6.78 | 6.25 | 5.37 | 14.02 | 7.88 | 6.57 | 6.15 | 5.19 |
| *G274* | 14.43 | 8.38 | 6.25 | 5.40 | 4.67 | 14.60 | 8.43 | 6.34 | 5.38 | 4.64 |
| *G28* | 11.28 | 6.52 | 4.82 | 4.11 | 3.43 | 11.50 | 6.45 | 4.75 | 4.13 | 3.60 |
| *G289* | 13.34 | 7.94 | 6.00 | 5.00 | 4.36 | 13.17 | 7.85 | 5.94 | 5.12 | 4.46 |
| *G300* | 5.50 | 4.10 | 2.77 | 2.22 | 1.62 | 5.19 | 4.00 | 2.64 | 2.23 | 1.76 |
| *G314* | 15.56 | 9.86 | 7.07 | 5.97 | 4.66 | 15.49 | 9.72 | 6.96 | 5.83 | 4.56 |
| *G33* | 15.57 | 10.01 | 7.29 | 6.28 | 5.01 | 15.07 | 9.64 | 6.97 | 6.06 | 4.83 |
| *G34* | 21.94 | 15.14 | 11.53 | 9.74 | 7.38 | 21.89 | 14.84 | 11.22 | 9.51 | 7.16 |
| *G348* | 19.46 | 11.59 | 8.54 | 7.30 | 6.09 | 19.30 | 11.48 | 8.37 | 7.13 | 5.97 |
| *G393* | 19.61 | 11.90 | 8.52 | 6.91 | 5.34 | 19.65 | 11.78 | 8.35 | 6.72 | 5.20 |
| *G397* | 11.63 | 6.86 | 5.22 | 4.38 | 3.89 | 11.69 | 6.79 | 5.07 | 4.42 | 3.84 |
| *G404* | 14.08 | 9.09 | 3.53 | 2.53 | 0.00 | 12.05 | 1.27 | 3.41 | 2.02 | 2.35 |
| *G417* | 13.19 | 7.50 | 5.20 | 4.39 | 3.86 | 13.13 | 7.48 | 5.30 | 4.42 | 3.74 |
| *G427* | 10.09 | 7.33 | 4.73 | 3.74 | 2.89 | 10.18 | 7.18 | 4.64 | 3.71 | 2.96 |
| *G43* | 11.27 | 6.93 | 4.65 | 3.92 | 3.25 | 11.19 | 6.82 | 4.53 | 3.85 | 3.19 |
| *G472* | 13.86 | 9.13 | 5.98 | 4.88 | 3.77 | 13.84 | 9.02 | 5.91 | 4.84 | 3.84 |
| *G48* | 11.10 | 6.85 | 5.32 | 4.77 | 3.99 | 10.94 | 6.71 | 5.15 | 4.67 | 3.92 |
| *G507* | 9.23 | 6.56 | 4.67 | 3.93 | 3.05 | 9.37 | 6.56 | 4.65 | 3.87 | 3.08 |
| *G533* | 15.10 | 9.63 | 6.46 | 5.27 | 4.14 | 15.04 | 9.50 | 6.40 | 5.28 | 4.29 |
| *G658* | 5.32 | 4.10 | 2.79 | 2.31 | 1.78 | 5.25 | 4.02 | 2.78 | 2.25 | 1.89 |
| *G669* | 14.49 | 10.38 | 7.32 | 6.79 | 5.30 | 14.51 | 10.27 | 7.17 | 6.70 | 5.21 |
| *G708* | 20.90 | 13.04 | 9.87 | 8.26 | 6.50 | 20.63 | 12.84 | 9.65 | 8.11 | 6.33 |
| *G712* | 17.89 | 13.29 | 11.49 | 10.46 | 8.77 | 17.77 | 13.16 | 11.28 | 10.44 | 8.66 |
| *G722* | 4.62 | 3.26 | 2.15 | 1.74 | 1.51 | 4.71 | 3.35 | 2.19 | 1.81 | 1.49 |
| *G730* | 8.25 | 2.48 | 1.77 | 1.54 | 1.21 | 8.11 | 2.45 | 1.73 | 1.56 | 1.19 |
| *G738* | 10.26 | 7.22 | 5.80 | 5.08 | 3.97 | 9.90 | 6.93 | 5.41 | 4.94 | 3.94 |
| *G749* | 9.34 | 6.30 | 3.99 | 3.33 | 2.63 | 9.24 | 6.11 | 3.88 | 3.28 | 2.65 |
| *G750* | 13.80 | 8.73 | 7.05 | 6.49 | 5.57 | 13.70 | 8.66 | 6.89 | 6.47 | 5.47 |
| *G860* | 13.02 | 7.58 | 6.30 | 5.94 | 5.52 | 13.01 | 7.56 | 6.24 | 5.92 | 5.45 |
| *G864* | 11.44 | 6.91 | 5.53 | 4.95 | 4.29 | 11.20 | 6.73 | 5.34 | 4.85 | 4.22 |
| *G870* | 12.15 | 6.91 | 5.26 | 4.58 | 3.73 | 11.83 | 6.64 | 4.99 | 4.36 | 3.64 |
| *G876* | 10.12 | 5.62 | 4.39 | 4.01 | 3.12 | 10.00 | 5.47 | 4.28 | 3.91 | 3.03 |
| *G896* | 8.35 | 4.58 | 3.36 | 2.91 | 2.55 | 8.23 | 4.60 | 3.36 | 2.92 | 2.55 |
| *G911* | 11.46 | 5.93 | 4.19 | 3.41 | 2.86 | 11.53 | 5.92 | 4.20 | 3.46 | 2.90 |
| *G912* | 13.66 | 7.43 | 6.10 | 5.34 | 4.68 | 13.87 | 7.53 | 6.12 | 5.37 | 4.71 |
| *G914* | 17.21 | 10.57 | 8.97 | 8.72 | 8.06 | 17.14 | 10.50 | 8.93 | 8.81 | 7.95 |
| *G936* | 14.68 | 8.30 | 7.00 | 6.24 | 5.71 | 14.60 | 8.30 | 6.93 | 6.28 | 5.67 |
| *G939* | 9.32 | 5.25 | 3.68 | 3.13 | 2.47 | 9.19 | 5.25 | 3.62 | 3.10 | 2.46 |
| *G942* | 11.79 | 6.37 | 4.31 | 3.54 | 2.98 | 11.68 | 6.34 | 4.31 | 3.52 | 3.01 |
| *G943* | 14.24 | 9.69 | 7.34 | 6.82 | 5.33 | 14.35 | 9.23 | 7.37 | 6.64 | 5.16 |
| *G951* | 12.33 | 7.10 | 5.74 | 4.92 | 4.42 | 12.43 | 7.19 | 5.75 | 5.06 | 4.41 |
| *G973* | 15.61 | 9.37 | 6.51 | 5.32 | 4.34 | 15.32 | 9.15 | 6.32 | 5.20 | 4.23 |
| *G978* | 6.14 | 4.12 | 2.86 | 2.24 | 1.78 | 5.72 | 4.15 | 2.74 | 2.26 | 1.83 |

Shown are the frequencies of cytosine to thymine mutations per position from the 5'-ends and the frequencies of guanine to adenine mutations per position from the 3'-ends. **IID:** individual ID; **DMG:** damage.

Table 10: Results of the *de novo* assembly using SPAdes

| ***IID*** | ***N*** | ***min*** | ***median*** | ***mean*** | ***N50*** | ***max*** | ***sum*** |
| --- | --- | --- | --- | --- | --- | --- | --- |
| *G34* | 113 | 124 | 19598 | 28379 | 47448 | 215826 | 3206892 |
| *G154* | 142 | 122 | 12198 | 22628 | 47897 | 189875 | 3213235 |
| *G404* | 210 | 122 | 7266 | 15309 | 35643 | 124759 | 3214970 |
| *G427* | 227 | 122 | 666 | 14217 | 44864 | 194626 | 3227419 |
| *G507* | 269 | 122 | 355 | 12032 | 47293 | 194642 | 3236675 |
| *G533* | 228 | 122 | 1511 | 14146 | 39078 | 220125 | 3225464 |
| *G722* | 204 | 122 | 3035 | 15800 | 47146 | 194633 | 3223209 |
| *G749* | 289 | 122 | 234 | 11209 | 44864 | 222852 | 3239506 |
| *G1083* | 272 | 124 | 8439 | 11747 | 17443 | 69059 | 3195389 |
| *G1149* | 141 | 212 | 16465 | 22712 | 39682 | 133277 | 3202523 |

**IID:** individual ID; **N:** number of assembled contigs; **min:** shortest contig; **median:** median of assembled contigs; **mean:** mean of assembled contigs; **N50:** N50 value for all assembled contigs; **max:** longest contig; **sum:** total length of produced bases.

Table 11: Coverage calculation using Mauve

| ***IID*** | ***coverage [%]*** | ***number of contigs used*** |
| --- | --- | --- |
| *G34* | 97.75 | 103 |
| *G154* | 97.93 | 102 |
| *G404* | 97.90 | 151 |
| *G427* | 97.79 | 112 |
| *G507* | 97.47 | 112 |
| *G533* | 97.88 | 119 |
| *G722* | 97.83 | 114 |
| *G749* | 97.76 | 113 |
| *G1083* | 97.68 | 269 |
| *G1149* | 97.80 | 136 |

**IID:** individual ID; **coverage:** percent of the reference genome covered by the assembled contigs; **number of contigs used:** total number of contigs used from the assembly to achieve the coverage stated in column two.

Table 12: *De novo* assembly using Megahit

| ***IID*** | ***N*** | ***min*** | ***median*** | ***mean*** | ***N50*** | ***max*** | ***sum*** |
| --- | --- | --- | --- | --- | --- | --- | --- |
| *G34* | 692210 | 200 | 381 | 707 | 806 | 192307 | 489.5e6 |
| *G154* | 487860 | 200 | 381 | 698 | 780 | 179644 | 340.5e6 |
| *G404* | 658240 | 200 | 446 | 878 | 1229 | 511145 | 578.4e6 |
| *G427* | 417175 | 200 | 481 | 1154 | 2424 | 430602 | 481.5e6 |
| *G507* | 1350424 | 200 | 387 | 537 | 485 | 264560 | 725.7e6 |
| *G533* | 97001 | 200 | 400 | 827 | 1288 | 276622 | 80.27e6 |
| *G722* | 880484 | 200 | 389 | 740 | 878 | 437126 | 651.6e6 |
| *G749* | 52084 | 200 | 457 | 978 | 1432 | 307085 | 50.97e6 |
| *G1083* | 363033 | 200 | 502 | 1164 | 2298 | 306664 | 422.6e6 |
| *G1149* | 378579 | 200 | 468 | 1022 | 1709 | 493357 | 387.2e6 |

**IID:** individual ID; **N:** Number of assembled contigs; **min:** shortest contig; **median:** Median of assembled contigs; **mean:** Mean of assembled contigs; **N50:** N50 Value for all assembled contigs; **max:** longest contig; sum: total length of produced bases.

Table 13: Coverage calculation using Mauve based on pre-processed data

| ***IID*** | ***coverage [%]*** | ***number of contigs used*** |
| --- | --- | --- |
| *G34* | 97.78 | 134 |
| *G154* | 97.67 | 120 |
| *G404* | 97.72 | 278 |
| *G427* | 97.67 | 128 |
| *G507* | 97.24 | 571 |
| *G533* | 97.27 | 280 |
| *G722* | 97.59 | 136 |
| *G749* | 97.52 | 133 |
| *G1083* | 97.17 | 1137 |
| *G1149* | 97.71 | 416 |

**IID:** individual ID; **coverage:** Percent of the reference genome covered by the assembled contigs; **number of contigs used:** total number of contigs used from the assembly to achieve the coverage stated in column two.

**Table 14: Allele call success per locus for 68 aDNA samples**

| ***Allele resolution*** | ***HLA-DRB1*** | ***HLA-DQB1*** | ***HLA-DPB1*** |
| --- | --- | --- | --- |
| *2-digit (serotype level)* | 82 | 96 | 46 |
| *- of these 4-digit (amino acid level)* | 57 | 79 | 46 |
| *NA (no call possible)* | 56 | 41 | 92 |

NA = no data availableTable 15: Individual allele call at HLA class II genes

| ***IID*** | ***HLA-DRB1_1*** | ***HLA-DRB1_2*** | ***HLA-DQB1_1*** | ***HLA-DQB1_2*** | ***HLA-DPB1_1*** | ***HLA-DPB1_2*** |
| --- | --- | --- | --- | --- | --- | --- |
| *G21* | DRB1*04:01 | DRB1*13:01 | DQB1*06:03 | DQB1*03:02 | DPB1*131:01 | NA |
| *G022* | NA | NA | NA | NA | NA | NA |
| *G24* | DRB1*07:01 | DRB1*08:01 | DQB1*04 | DQB1*03:03 | NA | NA |
| *G28* | NA | NA | DQB1*06:02 | NA | DPB1*04:01 | NA |
| *G33* | DRB1*13:01 | DRB1*15 | DQB1*06:02 | DQB1*06:03 | NA | NA |
| *G34* | DRB1*13:01 | DRB1*16:02 | DQB1*06:03 | DQB1*05:02 | DPB1*54:01 | DPB1*57:01 |
| *G43* | DRB1*04:04 | DRB1*15:01 | DQB1*03:02 | DQB1*06:02 | DPB1*03:01 | DPB1*46:01 |
| *G48* | DRB1*01:01 | DRB1*07:01 | DQB1*05:01 | DQB1*03:03 | DPB1*04:01 | DPB1*04:02 |
| *G102* | DRB1*04:03 | DRB1*11 | DQB1*03:01 | DQB1*03:02 | DPB1*04:02 | NA |
| *G104* | DRB1*04 | NA | NA | NA | DPB1*422:01 | NA |
| *G117* | NA | NA | NA | NA | NA | NA |
| *G118* | NA | NA | NA | NA | NA | NA |
| *G119* | DRB1*15:01 | DRB1*15:01 | DQB1*06:02 | DQB1*06:02 | DPB1*04:01 | DPB1*240:01 |
| *G120* | DRB1*13 | DRB1*15:01 | DQB1*06:02 | DQB1*06:04 | DPB1*03:01 | NA |
| *G131* | NA | NA | NA | NA | NA | NA |
| *G140* | NA | NA | NA | NA | NA | NA |
| *G149* | NA | NA | NA | NA | NA | NA |
| *G154* | DRB1*04 | DRB1*15 | DQB1*06:02 | DQB1*03:02 | NA | NA |
| *G164* | NA | NA | NA | NA | NA | NA |
| *G165* | DRB1*04:01 | DRB1*15 | DQB1*06:02 | DQB1*03:02 | NA | NA |
| *G166* | NA | NA | NA | NA | NA | NA |
| *G189* | DRB1*04 | DRB1*07:01 | NA | NA | NA | NA |
| *G208* | DRB1*01:01 | DRB1*04 | DQB1*03:02 | DQB1*05:01 | DPB1*04:01 | NA |
| *G255* | NA | NA | NA | NA | NA | NA |
| *G274* | NA | NA | NA | NA | NA | NA |
| *G289* | NA | NA | NA | NA | NA | NA |
| *G300* | DRB1*03:01 | DRB1*15:01 | DQB1*02:01 | DQB1*06:02 | DPB1*04:01 | NA |
| *G314* | DRB1*04:01 | DRB1*09:01 | DQB1*03:02 | DQB1*03:03 | DPB1*04:01 | DPB1*258:01 |
| *G348* | DRB1*01 | DRB1*15 | DQB1*05:01 | DQB1*06:02 | DPB1*52:01 | DPB1*442:01 |
| *G393* | DRB1*03:01 | DRB1*04:01 | DQB1*03:01 | DQB1*02:01 | DPB1*04:01 | DPB1*20:01 |
| *G397* | DRB1*04:01 | DRB1*10 | DQB1*03:02 | DQB1*05:01 | NA | NA |
| *G404* | NA | NA | DQB1*02:01 | DQB1*03:02 | NA | NA |
| *G417* | NA | NA | DQB1*06 | NA | NA | NA |
| *G427* | NA | NA | NA | NA | NA | NA |
| *G472* | NA | NA | NA | NA | NA | NA |
| *G507* | DRB1*11 | DRB1*13:02 | DQB1*03:01 | DQB1*06:04 | NA | NA |
| *G533* | DRB1*04 | DRB1*15 | DQB1*03:02 | DQB1*06 | NA | NA |
| *G658* | NA | NA | DQB1*02 | DQB1*06:02 | DPB1*02:02 | NA |
| *G669* | DRB1*15:01 | DRB1*16:01 | DQB1*06:02 | DQB1*05:02 | DPB1*04:01 | NA |
| *G708* | DRB1*04:01 | DRB1*07 | DQB1*03:02 | DQB1*03:02 | DPB1*04:01 | DPB1*234:01 |
| *G712* | DRB1*03:01 | DRB1*15:01 | DQB1*02:01 | DQB1*06:02 | DPB1*16:01 | NA |
| *G722* | DRB1*15 | NA | DQB1*03 | DQB1*06:02 | NA | NA |
| *G730* | DRB1*01:01 | DRB1*15:01 | DQB1*05:01 | DQB1*06:02 | DPB1*04:01 | NA |
| *G738* | NA | NA | DQB1*03 | DQB1*06 | NA | NA |
| *G749* | DRB1*15 | NA | DQB1*06 | NA | DPB1*169:02 | NA |
| *G750* | DRB1*01 | NA | DQB1*05:01 | DQB1*06 | NA | NA |
| *G860* | DRB1*03 | DRB1*03 | DQB1*02:01 | DQB1*02:01 | DPB1*04:01 | DPB1*04:02 |
| *G864* | DRB1*03:01 | DRB1*13:02 | DQB1*02:01 | DQB1*06:04 | DPB1*03:01 | NA |
| *G870* | NA | NA | DQB1*02:01 | DQB1*06:02 | NA | NA |
| *G876* | DRB1*04:01 | DRB1*14:01 | DQB1*03:02 | DQB1*05:03 | DPB1*90:01 | NA |
| *G896* | DRB1*03:01 | DRB1*15:01 | DQB1*02:01 | DQB1*06:02 | DPB1*138:01 | NA |
| *G911* | DRB1*04:01 | DRB1*04:01 | DQB1*03:01 | DQB1*03 | DPB1*04:01 | NA |
| *G912* | DRB1*07 | DRB1*09 | DQB1*02:01 | DQB1*03:03 | DPB1*11:01 | NA |
| *G914* | NA | NA | DQB1*02 | DQB1*05:01 | DPB1*109:01 | DPB1*136:01 |
| *G936* | DRB1*04:04 | DRB1*15:01 | DQB1*03:02 | DQB1*06:02 | DPB1*04:01 | NA |
| *G939* | DRB1*01:01 | DRB1*01:01 | NA | NA | DPB1*04:01 | NA |
| *G942* | DRB1*04:03 | DRB1*15:01 | DQB1*03:10 | DQB1*06:02 | DPB1*16:01 | DPB1*442:01 |
| *G943* | NA | NA | NA | NA | NA | NA |
| *G951* | DRB1*04:01 | DRB1*04:01 | DQB1*03:01 | DQB1*03:02 | NA | NA |
| *G973* | DRB1*13:01 | DRB1*13:01 | DQB1*06:03 | DQB1*06:04 | DPB1*03:01 | NA |
| *G978* | NA | NA | DQB1*02 | DQB1*06 | NA | NA |
| *G1042* | NA | NA | NA | NA | NA | NA |
| *G1044* | NA | NA | DQB1*03:02 | DQB1*06 | DPB1*03:01 | NA |
| *G1049* | DRB1*14:01 | DRB1*15:01 | DQB1*05:03 | DQB1*06:02 | DPB1*452:01 | NA |
| *G1065* | DRB1*01:01 | DRB1*04:01 | DQB1*03:01 | DQB1*05:01 | DPB1*04:01 | NA |
| *G1083* | DRB1*01 | DRB1*08 | DQB1*04 | DQB1*05 | NA | NA |
| *G1137* | NA | NA | DQB1*02 | NA | NA | NA |
| *G1149* | DRB1*04:01 | DRB1*15:01 | DQB1*03:01 | DQB1*06:02 | DPB1*04:01 | NA |

NA = no data available

Table 16: Assignment of genetic sex

| ***IID*** | ***Reads on chrX*** | ***Reads on chrY*** | ***d_X_*** | ***d_Y_*** | ***r*** | ***HTS Sex*** | ***osteological sex*** |
| --- | --- | --- | --- | --- | --- | --- | --- |
| *G34* | 6667128 | 283808 | 4.27E-02 | 4.96E-03 | 0.12 | f | f |
| *G102* | 846300 | 56433 | 5.42E-03 | 9.86E-04 | 0.18 | f | f |
| *G104* | 243133 | 15516 | 1.56E-03 | 2.71E-04 | 0.17 | f | f |
| *G117* | 30109 | 2437 | 1.93E-04 | 4.26E-05 | 0.22 | f | f |
| *G119* | 861981 | 75634 | 5.52E-03 | 1.32E-03 | 0.24 | f | f |
| *G120* | 1042127 | 76520 | 6.68E-03 | 1.34E-03 | 0.20 | f | f |
| *G131* | 16010 | 645 | 1.03E-04 | 1.13E-05 | 0.11 | f | f |
| *G140* | 153980 | 11962 | 9.87E-04 | 2.09E-04 | 0.21 | f | f |
| *G149* | 110611 | 5157 | 7.09E-04 | 9.01E-05 | 0.13 | f | f |
| *G164* | 60145 | 4567 | 3.85E-04 | 7.98E-05 | 0.21 | f | f |
| *G165* | 791826 | 65051 | 5.07E-03 | 1.14E-03 | 0.22 | f | m |
| *G166* | 51182 | 2955 | 3.28E-04 | 5.16E-05 | 0.16 | f | f |
| *G189* | 339550 | 17590 | 2.18E-03 | 3.07E-04 | 0.14 | f | f |
| *G255* | 106688 | 8490 | 6.84E-04 | 1.48E-04 | 0.22 | f | f |
| *G274* | 49796 | 3286 | 3.19E-04 | 5.74E-05 | 0.18 | f | f |
| *G300* | 1308094 | 73356 | 8.38E-03 | 1.28E-03 | 0.15 | f | f |
| *G314* | 1564905 | 89448 | 1.00E-02 | 1.56E-03 | 0.16 | f | f |
| *G393* | 1749118 | 124011 | 1.12E-02 | 2.17E-03 | 0.19 | f | f |
| *G397* | 159951 | 11863 | 1.03E-03 | 2.07E-04 | 0.20 | f | f |
| *G417* | 257560 | 14832 | 1.65E-03 | 2.59E-04 | 0.16 | f | f |
| *G427* | 589705 | 20555 | 3.78E-03 | 3.59E-04 | 0.10 | f | f |
| *G472* | 1207645 | 51168 | 7.74E-03 | 8.94E-04 | 0.12 | f | f |
| *G507* | 4410208 | 154628 | 2.83E-02 | 2.70E-03 | 0.10 | f | f |
| *G533* | 359853 | 13038 | 2.31E-03 | 2.28E-04 | 0.10 | f | f |
| *G750* | 89239 | 5293 | 5.72E-04 | 9.25E-05 | 0.16 | f | f |
| *G870* | 168146 | 13396 | 1.08E-03 | 2.34E-04 | 0.22 | f | f |
| *G876* | 1490309 | 87957 | 9.55E-03 | 1.54E-03 | 0.16 | f | f |
| *G914* | 296151 | 18122 | 1.90E-03 | 3.17E-04 | 0.17 | f | ? |
| *G939* | 917890 | 61064 | 5.88E-03 | 1.07E-03 | 0.18 | f | f |
| *G942* | 607380 | 45333 | 3.89E-03 | 7.92E-04 | 0.20 | f | ? |
| *G951* | 174825 | 10062 | 1.12E-03 | 1.76E-04 | 0.16 | f | m |
| *G973* | 1860624 | 107088 | 1.19E-02 | 1.87E-03 | 0.16 | f | f |
| *G1042* | 22146 | 1357 | 1.42E-04 | 2.37E-05 | 0.17 | f | f |
| *G1044* | 361311 | 28402 | 2.32E-03 | 4.96E-04 | 0.21 | f | f |
| *G1049* | 696059 | 51460 | 4.46E-03 | 8.99E-04 | 0.20 | f | f |
| *G1065* | 2576933 | 158586 | 1.65E-02 | 2.77E-03 | 0.17 | f | f |
| *G1137* | 545693 | 21679 | 3.50E-03 | 3.79E-04 | 0.11 | f | f |
| *G1149* | 4254650 | 220118 | 2.73E-02 | 3.85E-03 | 0.14 | f | f |
| *G21* | 1166860 | 298748 | 7.48E-03 | 5.22E-03 | 0.70 | m | m |
| *G22* | 4075 | 1425 | 2.61E-05 | 2.49E-05 | 0.95 | m | f |
| *G24* | 557680 | 192628 | 3.57E-03 | 3.37E-03 | 0.94 | m | m |
| *G28* | 130012 | 41157 | 8.33E-04 | 7.19E-04 | 0.86 | m | m |
| *G33* | 679470 | 189406 | 4.35E-03 | 3.31E-03 | 0.76 | m | m |
| *G43* | 1607687 | 404617 | 1.03E-02 | 7.07E-03 | 0.69 | m | f |
| *G48* | 551461 | 173806 | 3.53E-03 | 3.04E-03 | 0.86 | m | f |
| *G118* | 24170 | 4834 | 1.55E-04 | 8.45E-05 | 0.55 | m | f |
| *G154* | 2426939 | 721380 | 1.56E-02 | 1.26E-02 | 0.81 | m | ? |
| *G208* | 2538587 | 679671 | 1.63E-02 | 1.19E-02 | 0.73 | m | m |
| *G289* | 8517 | 3115 | 5.46E-05 | 5.44E-05 | 1.00 | m | f |
| *G348* | 151389 | 46843 | 9.70E-04 | 8.19E-04 | 0.84 | m | m |
| *G404* | 440919 | 141731 | 2.83E-03 | 2.48E-03 | 0.88 | m | m |
| *G658* | 1102587 | 480343 | 7.07E-03 | 8.39E-03 | 1.19 | m | m |
| *G669* | 1755037 | 508688 | 1.12E-02 | 8.89E-03 | 0.79 | m | m |
| *G708* | 578913 | 147204 | 3.71E-03 | 2.57E-03 | 0.69 | m | m |
| *G712* | 470615 | 110958 | 3.02E-03 | 1.94E-03 | 0.64 | m | m |
| *G722* | 1305254 | 483536 | 8.36E-03 | 8.45E-03 | 1.01 | m | m |
| *G730* | 618527 | 147258 | 3.96E-03 | 2.57E-03 | 0.65 | m | m |
| *G738* | 100679 | 27665 | 6.45E-04 | 4.83E-04 | 0.75 | m | m |
| *G749* | 278145 | 100123 | 1.78E-03 | 1.75E-03 | 0.98 | m | f |
| *G860* | 1175340 | 308061 | 7.53E-03 | 5.38E-03 | 0.71 | m | ? |
| *G864* | 641931 | 176195 | 4.11E-03 | 3.08E-03 | 0.75 | m | m |
| *G896* | 365680 | 105145 | 2.34E-03 | 1.84E-03 | 0.78 | m | m |
| *G911* | 329532 | 77762 | 2.11E-03 | 1.36E-03 | 0.64 | m | m |
| *G912* | 154908 | 35725 | 9.93E-04 | 6.24E-04 | 0.63 | m | m |
| *G936* | 666639 | 145033 | 4.27E-03 | 2.53E-03 | 0.59 | m | m |
| *G943* | 63424 | 13175 | 4.06E-04 | 2.30E-04 | 0.57 | m | M |
| *G978* | 129595 | 52728 | 8.31E-04 | 9.21E-04 | 1.11 | m | M |
| *G1083* | 403229 | 133174 | 2.58E-03 | 2.33E-03 | 0.90 | m | ? |
|  |  |  |  |  |  |  |  |

**IID:** individual ID; **m**: male; **f**: female; **?**: sex determination was not possible

References

1. Pedersen, D. D. *The presence of tuberculosis in Danish skeletons AD 800-1800 – from skeletal data to paleoepidemiological analyses*. (2016).
2. Boldsen, J. L., Rasmussen, K. L., Riis, T., Dittmar, M. & Weise, S. Schleswig: medieval leprosy on the boundary between Germany and Denmark. *Anthropol. Anzeiger* **70,** 273–87 (2013).
3. Boldsen, J. L. Leprosy in Medieval Denmark—Osteological and epidemiological analyses. *Anthropol. Anz.* **67**, 407-425 (2009).
4. Boldsen, J. L. & Mollerup, L. Outside St. Jørgen: Leprosy in the medieval Danish city of Odense. *Am. J. Phys. Anthropol.* **130**, 344-351 (2006).
5. Arentoft, E. *De spedalskes hospital : udgravninger af Sankt Jørgensgården i Odense*. (Odense Bys Museer, 1999).
6. Jantzen, C., Madsen, P. K. & Kieffer-Olsen, J. De små brødre - Udgravning af gråbrødrekloster i Ribe. *Skalk* **2,** 11–16 (1995).
7. Andersen, L. Ribe Gråbrødrekloster - det sidste kapitel. *Mark og montre* **39,** 23–40 (2003).
8. Madsen, P. K., Jørgensen, J. B. & Petersen, S. G. Udgravninger på Sct. Catharinæ kloster i Ribe. *Mark og montre* **20,** 59–69 (1984).
9. Madsen, H. A. & Søvsø, M. Liv og død i Ribe omkring år 1700 – de gravlagte i processionsgangen syd for Ribe Domkirke. *By, Marsk og Geest* **22,** 56–75 (2010).
10. Søvsø, M. I hjertet af Ribe. *Skalk* **4,** 3–9 (2009).
11. Kieffer-Olsen, J. *Grav og gravskik i det middelalderlige Danmark: 8 Kirkegårdsudgravninger*. (1993).
12. Kieffer-Olsen, J., Boldsen, J. & Pentz, P. *En nyfunden kirke ved Bygholm*. (Vejle Amts Årbog, 1986).
13. Boldsen, J. L. Leprosy and mortality in the Medieval Danish village of Tirup. *Am. J. Phys. Anthropol.* **126,** 159–168 (2005).
14. Lüdtke, H. in *Ausgrabungen in Schleswig. Berichte und Studien Band 12* 9–84 (Wachholtz Verlag, 1997).
15. White, T. D. & Folkens, P. A. *The Human Bone Manual*. (Academic Press, 2005).
16. Milner, G. R. & Boldsen, J. L. Humeral and femoral head diameters in recent white American skeletons. *J. Forensic Sci.* **57,** 35–40 (2012).
17. Boldsen, J. L. *Leprosy in medieval Denmark: A comprehensive analysis*. (2007).
18. Schuenemann, V. J., et al. Genome-wide comparison of medieval and modern Mycobacterium leprae. *Science* **341**, 179-183 (2013).
19. Schäfer, C., Schmidt, A. H. & Sauter, J. Hapl-O-mat: A versatile software for haplotype frequency estimation. *HLA* **87,** 236–320 (2016).
20. Huson, D. H., Auch, A. F., Qi, J. & Schuster, S. C. MEGAN analysis of metagenomic data. *Genome Res.* **17,** 377-386 (2007).
21. Felsenstein, J. Confidence Limits on Phylogenies: An Approach Using the Bootstrap. *Evolution (N. Y).* **39,** 783 (1985).
22. Nei, M. & Kumar, S. *Molecular Evolution and Phylogenetics*. (Oxford University Press, 2000).
23. Kumar, S., Stecher, G. & Tamura, K. MEGA7: Molecular Evolutionary Genetics Analysis Version 7.0 for bigger datasets. *Mol. Biol. Evol.* **33,** 1870-1874 (2016).
24. Tamura, K. Estimation of the number of nucleotide substitutions when there are strong transition-transversion and G+C-content biases. *Mol. Biol. Evol.* **9,** 678–87 (1992).
25. Saitou, N. & Nei, M. The neighbor-joining method: a new method for reconstructing phylogenetic trees. *Mol. Biol. Evol.* **4,** 406–25 (1987).
26. Hall, T. A. BioEdit: a user-friendly biological sequence alignment editor and analysis program for Windows 95/98/NT. *Nucleic Acids Symp. Ser.* **41,** 95-98 (1999).
