# Supplementary material for: Ancient DNA study reveals HLA susceptibility locus for leprosy in medieval Europeans
Source: Nat Commun. 2018 May 1;9:1569. doi: 10.1038/s41467-018-03857-x (PMC5931558; doi:10.1038/s41467-018-03857-x)
Supplement: Supplementary file 3 — Description of Additional Supplementary Files [file 41467_2018_3857_MOESM3_ESM.pdf]

Description of Additional Supplementary Files

File Name: Supplementary Data 1
Description: Sample overview per graveyard

File Name: Supplementary Data 2
Description: List of all SNPs in the 16 M. leprae genomes.

File Name: Supplementary Data 3
Description: Phylogenetic Models

File Name: Supplementary Data 4
Description: IBD estimation
